# Gene Expression Profiling of Multiple Sclerosis Pathology Identifies Early Patterns of Demyelination Surrounding Chronic Active Lesions

**DOI:** 10.3389/fimmu.2017.01810

**Published:** 2017-12-21

**Authors:** Debbie A. E. Hendrickx, Jackelien van Scheppingen, Marlijn van der Poel, Koen Bossers, Karianne G. Schuurman, Corbert G. van Eden, Elly M. Hol, Jörg Hamann, Inge Huitinga

**Affiliations:** ^1^Neuroimmunology Research Group, Netherlands Institute for Neuroscience, An Institute of the Royal Netherlands Academy of Arts and Sciences, Amsterdam, Netherlands; ^2^Neurodegeneration Research Group, Netherlands Institute for Neuroscience, An Institute of the Royal Netherlands Academy of Arts and Sciences, Amsterdam, Netherlands; ^3^Department of Translational Neuroscience, Brain Center Rudolf Magnus, University Medical Center Utrecht, Utrecht, Netherlands; ^4^Swammerdam Institute for Life Sciences, Center for Neuroscience, University of Amsterdam, Amsterdam, Netherlands; ^5^Department of Experimental Immunology, Academic Medical Center, University of Amsterdam, Amsterdam, Netherlands

**Keywords:** multiple sclerosis, microarray, active lesions, microglia, demyelination, scavenger receptor, lipid uptake

## Abstract

In multiple sclerosis (MS), activated microglia and infiltrating macrophages phagocytose myelin focally in (chronic) active lesions. These demyelinating sites expand in time, but at some point turn inactive into a sclerotic scar. To identify molecular mechanisms underlying lesion activity and halt, we analyzed genome-wide gene expression in rim and peri-lesional regions of chronic active and inactive MS lesions, as well as in control tissue. Gene clustering revealed patterns of gene expression specifically associated with MS and with the presumed, subsequent stages of lesion development. Next to genes involved in immune functions, we found regulation of novel genes in and around the rim of chronic active lesions, such as *NPY, KANK4, NCAN, TKTL1*, and *ANO4*. Of note, the presence of many foamy macrophages in active rims was accompanied by a congruent upregulation of genes related to lipid binding, such as *MSR1, CD68, CXCL16*, and *OLR1*, and lipid uptake, such as *CHIT1, GPNMB*, and *CCL18*. Except *CCL18*, these genes were already upregulated in regions around active MS lesions, showing that such lesions are indeed expanding. *In vitro* downregulation of the scavenger receptors *MSR1* and *CXCL16* reduced myelin uptake. In conclusion, this study provides the gene expression profile of different aspects of MS pathology and indicates that early demyelination, mediated by scavenger receptors, is already present in regions around active MS lesions. Genes involved in early demyelination events in regions surrounding chronic active MS lesions might be promising therapeutic targets to stop lesion expansion.

## Introduction

Multiple sclerosis (MS) is a neurological disease characterized by focal demyelinating lesions in the central nervous system, leading to a variety of symptoms, including problems with motor control, numbness or tingling sensation, cognitive problems, depression, and fatigue. Both genetic and environmental factors play a role in the onset and progression of MS [reviewed in Ref. (1, 2)]. Demyelination in MS is mediated by activated microglia and infiltrating macrophages, and in brains and the spinal cord of MS patients, both (chronic) active lesions and inactive scars are found. It is not clear why MS lesions are active demyelinating and which mechanisms contribute to the halt of lesion activity.

Depending on the level of demyelination and microglia/macrophage activation, MS lesions are characterized as active, chronic active, or inactive (3, 4). Active lesions contain lipid-laden microglia/macrophages throughout the lesions, while chronic active MS lesions have a demyelinated sclerotic core, surrounded by a rim of foamy microglia/macrophages. A recent magnetic resonance imaging study showed that chronic active lesions expand in time (5), and it is thought that at some point, active lesions turn into inactive sclerotic scars. Moreover, we found that chronic active lesion load correlates with fast progression of the disease, illustrating the clinical implications of lesion expansion (Luchetti et al., submitted).

Identification of gene expression in presumed, subsequent stages of MS lesions will increase insight into the molecular mechanisms related to lesion activity and halt. Gene expression profiling studies so far, on tissue blocks containing MS lesions from limited numbers (3–5) of MS patients per study, showed overall upregulation of pro-inflammatory pathways (6–10) and oxidative injury (11). One gene expression analysis of normal appearing white matter (NAWM) in MS demonstrated upregulation of genes associated with immunosuppression and protective mechanisms, but also pro-inflammatory mechanisms, suggesting a state of low-level inflammation and an unsteady balance (12, 13).

Previously, we analyzed differential gene expression between rims and regions surrounding chronic active and inactive MS lesions in substantial numbers of well-characterized MS brain donors by quantitative polymerase chain reaction (qPCR) and identified downregulation of macrophage inhibitory molecules around chronic active lesions (13). In this follow-up study, we set out a hypothesis-free microarray approach to study gene expression in rims and peri-rim regions of and around chronic active and inactive MS lesions from 15 MS patients and white matter (WM) of 10 matched control subjects. We identified gene expression specifically related to MS and to the assumed, subsequent stages of lesion development. Strikingly, genes connected with lipid binding and uptake were increased in the rim and peri-rim of chronic active lesions.

## Materials and Methods

### Human Tissue

Post-mortem human brain tissue was provided by the Netherlands Brain Bank (NBB, Amsterdam, The Netherlands^1^). Informed consent was obtained from donors for brain autopsy and the use of tissue and clinical information for research purposes. At the time of death, 12 patients had relapsing-remitting course of the disease, 1 had a primary-progressive disease course, and for 2, the disease course could not be determined. MS diagnosis was confirmed post-mortem by a neuropathologist. One-way ANOVA analysis (Kruskal–Wallis test) showed no significant difference in age, post-mortem delay, or pH of cerebrospinal fluid (CSF) between the groups. Detailed donor characteristics are provided in Table 1; Table S1 in Supplementary Material.

**Table 1:** Donor characteristics per group.

Tissue type	Lesion area	Sex	Age	pH	PMD	RIN
Chronic active	Rim	2 M/5 F	49.4 ± 8.6	6.42 ± 0.17	8:24 ± 1:55	6.39 ± 0.67
	PL-NAWM					6.43 ± 0.33
Inactive	Rim	2 M/6 F	63.3 ± 11.7	6.43 ± 0.21	9:03 ± 0:45	5.79 ± 0.62
	PL-NAWM					6.16 ± 0.50
Control		3 M/7 F	59.7 ± 10.4	6.67 ± 0.35	8:23 ± 2:51	7.42 ± 0.67
One-way ANOVA			0.1351	0.3941	0.6958	0.0003

*Age, age at death (years); F, female; M, male; pH, pH of CSF; PL-NAWM, peri-lesional normal appearing white matter; PMD, post-mortem delay (h:min); RIN, RNA integrity number*.

*Data are represented as mean ± SD*.

### Tissue Dissection and RNA Isolation

Cryostat sections were stained for myelin proteolipid protein (PLP; Serotec, Oxford, UK) and HLA-DP/Q/R (DAKO, Glostrup, Denmark) to assess MS lesion activity. Chronic active MS lesions were characterized by a sclerotic hypocellular demyelinated core, surrounded by a clear distinct rim of foamy HLA-positive macrophages (14). Inactive MS lesions were sclerotic demyelinated lesions without activated macrophages (3). Frozen chronic active and inactive MS lesions were cut in 20-µm sections using a cryostat and mounted on PALM MembraneSlides (PALM Microlaser Technologies, Munich, Germany). Every fifth to seventh section was stained with Sudan Black to confirm the lesion was still present and to facilitate dissection. Furthermore, every first and last section was stained for PLP and HLA-D/Q/R to assure continuous lesion activity.

The rim and peri-lesional (PL)-NAWM were dissected by laser dissection microscopy and stored in ice-cold TRIsure (Bioline, London, UK). Control tissue was dissected inside the cryostat using a pre-chilled scalpel and also stored in ice-cold TRIsure. After addition of chloroform and centrifugation, the aqueous phase was removed and mixed with an equal volume of 70% RNase-free ethanol. Samples were then applied to an RNeasy Mini column (Qiagen, Valencia, CA, USA) and further processed according to manufacturer’s instructions. RNA yield was determined using a NanoDrop ND-1000 spectrophotometer (NanoDrop Technologies, Wilmington, DE, USA), and quality was assessed on a Bioanalyzer 2100 (Agilent Technologies, Palo Alto, CA, USA). Only samples with RNA integrity number (RIN) values ≥5 were included. In total, 7 chronic active MS lesions, 8 inactive MS lesions, and WM of 10 control donors were included in this study. RIN values of control donors were significantly higher than of any of the MS lesion subareas. However, there was no difference in RIN value between the rim of chronic active versus the rim of inactive MS lesions, or the PL-NAWM of chronic active versus the PL-NAWM of inactive MS lesions.

### Sample Preparation and Microarray Hybridization

The Low Input Quick Amp Labeling Kit (Agilent Technologies) was used for sample amplification and fluorescent labeling according to manufacturer’s instructions. Briefly, 100 ng experimental RNA input and 50 ng reference pool RNA input was used for linear amplification and fluorescent labeling. The reference pool RNA was extracted from snap-frozen tissue dissected from a diversity of anatomical regions from control and MS brains, including MS lesions and NAWM, as well from tonsil. Experimental samples were labeled with Cy5-CTP, and the reference pool was labeled with Cy3-CTP (Perkin Elmer, Waltham, MA, USA). The cRNA samples were purified using RNeasy mini columns (Qiagen), and quantity and labeling efficiency (specific activity) was determined on a NanoDrop.

Prior to hybridization, 825 ng Cy3- and Cy5-labeled cRNA samples were fragmented by 30 min incubation at 60°C in 1× fragmentation buffer (Agilent Technologies). Each time, one Cy5-labeled experimental sample and one Cy3-labeled reference pool sample were hybridized to an Agilent Human Gene Expression 4 × 44K v2 Microarray (Part Number G4845A) for 17 h at 65°C in a rotating hybridization chamber. Arrays were washed in 6× saline sodium phosphate-EDTA (SSPE)/0.005% *N*-lauroylsarcosine (Sigma-Aldrich, St. Louis, MO, USA) for 5 min, then in 0.06× SSPE/0.005% *N*-lauroylsarcosine for 1 min, and finally in acetonitrile (Sigma-Aldrich) for 30 s. After drying in a nitrogen flow, arrays were scanned using an Agilent DNA Microarray Scanner at 5 mm resolution and 100% photomultiplier tube setting. Microarray scans were quantified using Agilent Feature Extraction software (version 9.5.3.1).

### Microarray Normalization and Single Gene Analysis

Common reference cRNA was co-hybridized to every microarray slide to allow for accurate comparison of expression levels across different cDNA microarray experiments. In this way, a ratio between the experimental and reference material could be calculated for every spot, and expression levels across different hybridizations could be compared. Raw expression data were imported into the R statistical processing environment using the LIMMA package in Bioconductor.^2^ All features for which one or more foreground measurements were flagged as saturated or as a non-uniformity outlier by the feature extraction software were excluded from further analysis. As overall background levels were very low, no background correction was performed.

Data within an array were normalized using *loess* (LIMMA), which was followed by a between-array normalization using the *Gquantile* algorithm in LIMMA. Subsequently, for probes that mapped to the same gene, the average *M*- and *A*-value of those probes were used for further analyses.

Differential gene expression was assessed using a single channel analysis on the M-values using Bayesian statistics in LIMMA. Three contrasts were investigated: (I) chronic active rim vs. inactive rim, (II) chronic active PL-NAWM vs. inactive PL-NAWM, and (III) chronic active PL-NAWM vs. control. Correction for multiple testing was performed with the Benjamini–Hochberg algorithm. Genes with a *p*-value <0.05 were considered significant.

### Cluster Analysis of Gene Expression Data in Different Stages of Lesion Activity

**Figure 1:**
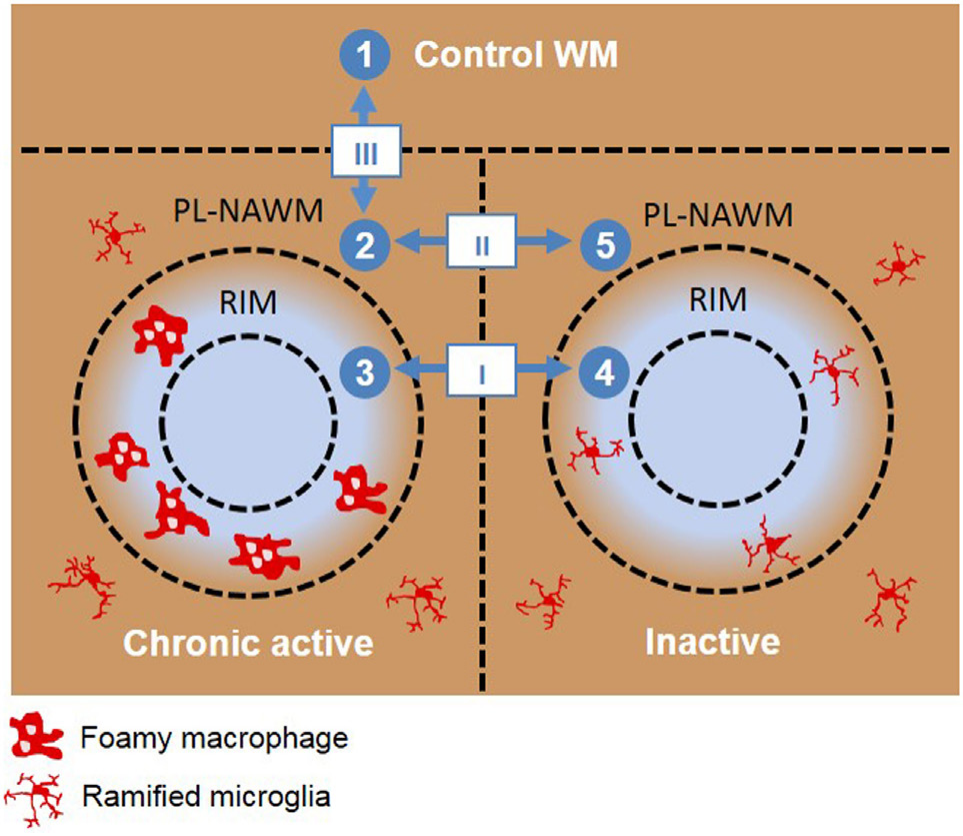
Schematic overview of the different microarray analyses done. Roman numbers indicate direct comparisons. (I) chronic active rim vs. inactive rim, (II) chronic active peri-lesional (PL)-normal appearing white matter (NAWM) vs. inactive PL-NAWM, and (III) control vs. chronic active PL-NAWM. Arabic numbers indicate the sequence used for cluster analysis. (1) Control white matter (WM), (2) chronic active PL-NAWM, (3) chronic active rim, (4) inactive rim, (5) inactive PL-NAWM.

In order to follow the expression of individual transcripts in different presumed subsequent stages of lesion activity and demyelination, expression profiles were constructed from regions that represent no pathology (control NAWM), the early events in demyelination (PL-NAWM around chronic active MS lesions), fully active demyelination (rim of chronic active MS lesions), halt of demyelination (rim of inactive MS lesions), and absence or suppression of early demyelination (PL-NAWM of inactive lesions, Figure 1). The NIA Array Analysis software was used to find these clusters of genes showing the same expression pattern across the different subareas studied (15). The intensity values of all genes were used as input. The NIA Array Analysis software uses ANOVA (with error variance averaging and Benjamini correction for false discovery rate) to test statistical significant genes. Only significant genes were displayed.

### Gene Ontology Overrepresentation Analysis

The overrepresentation of specific GO terms within the different clusters was analyzed using GOstat with the goa_human database (minimum path length of 3 and Benjamini correction for false discovery rate). All statistical significant genes per cluster were used as input, and all genes measured on the array were used as the background set of genes.

### cDNA Synthesis and qPCR

Reverse transcription was performed in a reaction mixture of 10 µl containing 100 ng RNA and gDNA Wipeout Buffer, incubated for 2 min at 42°C, and Quantiscript^®^ Reverse Transcriptase, Quantiscript Buffer, and room temperature (RT) Primer Mix (Qiagen Benelux, Venlo, The Netherlands), incubated for 15 min at 42°C. RT transcriptase was inactivated by incubation for 3 min at 95°C.

Primer pairs for real-time qPCR were designed using the NCBI primer basic local alignment search tool; see Table S2 in Supplementary Material for the primer pairs used in this study. Specificity was tested on cDNA derived from brain or laser dissection microscopy-isolated test brain tissue of MS donors and control donors by assessment of the dissociation curve and PCR product, as determined by size fractionation on an 8% sodium dodecyl sulfate polyacrylamide gel electrophoresis gel.

Quantitative polymerase chain reaction reactions were performed with SYBR Green PCR Master Mix (Applied Biosystems, Foster City, CA, USA) with samples containing equal cDNA concentrations of 2–3.5 ng total RNA per reaction. Analysis was performed according to the manufacturer’s protocol and the ABI Prism 7300 Sequence Detection System (Applied Biosystems). Target genes were normalized to the geometric mean of glyceraldehyde 3-phosphate dehydrogenase (*GAPDH*), tubulin α (*TUBA1A*), or elongation factor 1 alpha (*EEF1A1*) mRNA expression, which did not differ significantly between the different groups studied. Fold differences were calculated using the 2^−ΔΔCT^ method (16).

### Immunohistochemistry

Tissue of donors used for detection of protein expression are displayed in Table 1. For CHIT1, frozen sections (20 µm) of both active and inactive MS lesions and control tissue were fixed for 20 min in 4% paraformaldehyde. For GPNMB, OLR1, and ANO4, paraffin-embedded sections (6 µm) of both active and inactive MS lesions and control tissue were deparaffinized with xylene and rehydrated, and antigen retrieval was performed by incubation in tris-buffered saline (TBS) for GPNMB and OLR1 and in citrate buffer at pH6 for ANO4 (microwave, 10 min at 700 W). Aspecific binding was blocked by incubation in 10% normal horse serum (NHS) for 30 min at RT, followed by incubation with primary antibodies directed at CHIT1 (NBP1-84490, 1:20; Novus Biologicals, Abingdon, UK), GPNMB (MAB15501, 1:200; R&D Systems, Oxon, UK), OLR1 (H00004973-D01, 1:500; Abnova, Taoyuan City, Taiwan), or ANO4 (19488-1-AP, 1:50; Proteintech, Manchester, UK) diluted in incubation buffer (0.25% gelatin and 0.5% Triton-X in TBS, pH 7.6), for 1 h at RT. Immunoreactivity was visualized by using avidin–biotin complex (Vector PK-6100, Burlingame, CA, USA), followed by diaminobenzidine chromogenic substrate system (EnVision, DAKO) for CHIT1 or immediately by using the EnVision detection system (Dako) for GPNMB and OLR1. Sections were counterstained by 0.025% cresyl violet and embedded in Entellan. Immunoreactivity was examined using a Zeiss Axioskop 9801 light microscope (Zeiss, Oberkochen, Germany).

### Cell Culture and Gene Silencing

The human monocytic cell line THP-1 was cultured in Roswell Park Memorial Institute (RPMI) glutaMAX medium containing 10% fetal calf serum and 1% penicillin/streptomycin. For flow cytometric or PCR analysis, cells were cultured in plates coated with poly(2-hydroxyethyl methacrylate), otherwise known as hydron (Sigma-Aldrich), to prevent adherence. For immunocytochemistry, cells were cultured on glass coverslips. Cells were differentiated into macrophage-like cells by stimulation with 160 nM phorbol 12-myristate 13-acetate (PMA) for 24 h, followed by another 24 h culture in normal medium. To measure unlabeled myelin uptake over time, cells were stimulated with 8 nM PMA for 48 h, followed by 5 days culture in normal medium.

Gene silencing was performed using locked nucleic acid (LNA) oligonucleotides, designed using the siDesign center of Thermo Scientific and synthesized by Santaris Pharma A/S (Hørsholm, Denmark). The following oligonucleotide sequences and concentrations were used: *MSR1* (CCCGTGAGACTTTGAG; 2 µM), *CXCL16* (AGTGAGCTCTTTGTCC; 5 µM), *OLR1* (CTCATTCAGCTTCCGA; 2.5 µM), and *CD68* (AACTGAAGCTCTGCCC; 2.5 µM). The 16-mers contained three LNA moieties at both termini (underlined). Oligonucleotide uptake was achieved without any additives, through a process called gymnosis (17). Differentiated cells were incubated with the oligonucleotides for 6 days before myelin uptake was tested. For inhibition of a broad spectrum of scavenger receptors, cells were pre-incubated with 100, 500, or 1,000 µg/ml fucoidan (Sigma, Zwijndrecht, The Netherlands) for 45 min before myelin was added.

### Phagocytosis Assay

Myelin was isolated from the myelin-containing fraction of post-mortem human brain tissue collected after Percoll gradient separation. Unlabeled myelin was used to measure myelin uptake over time, and myelin stained with the pH sensitive dye pHrodo red (Invitrogen) was used for gene silencing experiments to visualize uptake in the lysosomal compartment, as described recently (18).

Free floating THP-1 macrophages were incubated with 12.5 µg pHrodo-labeled MS or control myelin per 80,000 cells for 24 h. After incubation, the cells were collected and washed in cold phosphate-buffered saline (PBS) with 1% bovine serum albumin for quantification of myelin uptake by flow cytometry after gene silencing. Expression of *CHIT1* and *GPNMB* was determined after incubation with unlabeled 12.5 µg MS or control myelin for 1, 2, or 5 days in duplo. After 5 days, the medium was refreshed and cells were incubated for 80 more hours in normal medium. Harvested cells were stored in TRisure and gene expression of *CHIT1* and *GPNMB* was analyzed by qRT-PCR.

For flow cytometric analysis, cells were incubated with the viability dye eFluor 780 (eBiosciences; 1:2,000) for 30 min on ice. Uptake of pHrodo-labeled myelin was measured on a FACSCanto machine (BD Biosciences) and analyzed using FlowJo 7.6 software (Figure S1 in Supplementary Material). Phagocytosis was expressed as percentage of live cells that took up myelin and as geomean fluorescence intensity of the pHrodo signal indicating the total amount of myelin phagocytosed.

For immunocytochemical analysis, cells on glass coverslips were fixed in 4% paraformaldehyde for 15 min and washed with PBS. Aspecific binding was blocked by incubation in 10% NHS for 30 min at RT, followed by incubation with the primary antibody directed at MSR1 (MAB1716, 1:100; Abnova), diluted in incubation buffer (0.25% gelatin and 0.5% Triton-X in TBS pH 7.6), o/n at 4°C. The next day, cells were washed and incubated with the fluorescently labeled secondary antibody (donkey anti-mouse Cy3 conjugated antibody, 1:1,000; Millipore) in incubation buffer with Hoechst 1:1,000 for nuclear staining for 1 h at RT. Coverslips were then washed in PBS and demineralized water and embedded in mounting medium (0.605 g Tris pH 8.5, 12.5 ml glycerol 100%, and 5 g Mowiol; EMD Chemicals, Gibbstown, NJ, USA).

Fluorescent images were taken on an Axiovert microscope (Zeiss) with Neoplanfluor objectives using an Exi Aqua Bio-imaging microscopy camera (QImaging, Surray, BC, Canada) and ImagePro software (MediaCybernetics, Bethesda, MD, USA).

### Statistical Analysis

Statistical analysis of qPCR validation and cell culture experiments was performed using GraphPad Prism version 6 software (GraphPad Inc., La Jolla, CA, USA). The non-parametric Kruskal–Wallis test, followed by *post hoc* comparisons (Mann–Whitney *U* test) was performed to assess the regulation of genes of interest and the effect of gene silencing and addition of fucoidan. Differences between THP-1 cells incubated with MS, control, or no myelin over time were assessed with One-way ANOVA, followed by Tukey’s multiple comparisons test. *p* values <0.05 were considered significant.

## Results

### Identification of Genes That Show Significantly Altered Expression in Different MS Lesion Subregions

**Figure 2:**
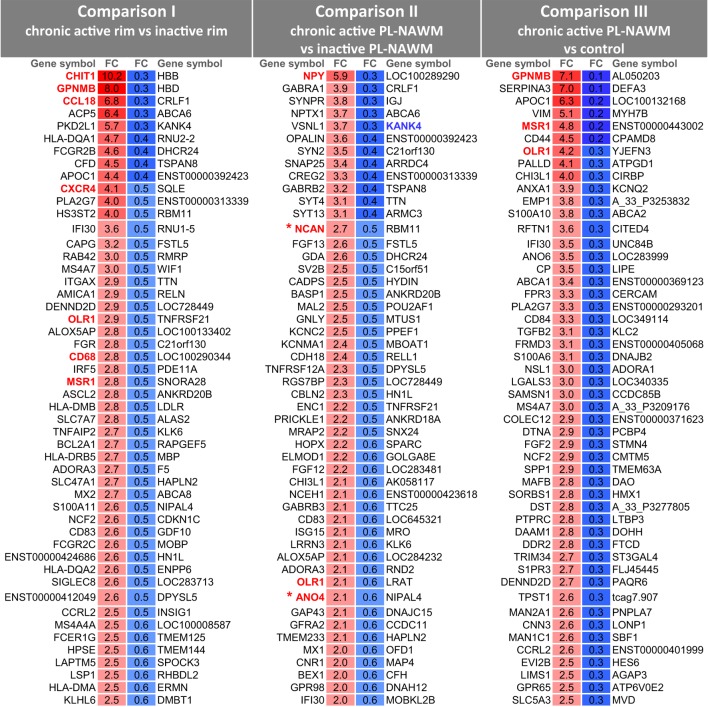
Top 50 significantly upregulated and downregulated genes in multiple sclerosis lesion subregions. For a description of the comparisons, see Figure 1 and text. Upregulated genes are indicated in red and downregulated genes are indicated in blue. Genes colored and marked in bold have been selected for further investigation. Genes expressed in cluster 3 are marked by an asterisk. Further details on the genes and *p* values are provided in Tables S3A–F in Supplementary Material. FC, fold change.

Using laser-based microdissection, we isolated the rim and PL region of and around chronic active and inactive MS lesions from 15 MS patients and WM of 10 matched control subjects and analyzed differences in gene expression using Agilent Human Gene Expression 4 × 44K v2 microarrays. The following comparisons were made: I. chronic active rim vs. inactive rim, (II) chronic active PL-NAWM vs. inactive PL-NAWM, and (III) chronic active PL-NAWM vs. control WM (Figure 1; Roman numbers). In comparison I, we expected to find genes involved in either active demyelination (upregulated in chronic active rim) or cessation of demyelination (upregulated in inactive rim). Genes in comparison II were expected to be involved in early demyelination and expansion of lesions (upregulated in chronic active PL-NAWM) or the prevention and exhaustion of lesion expansion (upregulated in inactive PL-NAWM). Finally, comparison III was expected to show genes involved in initial lesion onset (either protective genes that are downregulated or inflammatory or phagocytic genes that are upregulated in chronic active PL-NAWM). This resulted in a total of 1,251 significantly regulated genes in comparison I, 587 genes in comparison II, and 3,434 genes in comparison III, with a *p*-value <0.05. For an overview of the top 50 significantly upregulated and downregulated genes per comparison, see Figure 2; Tables S3A–F in Supplementary Material.

### Cluster Analysis

**Figure 3:**
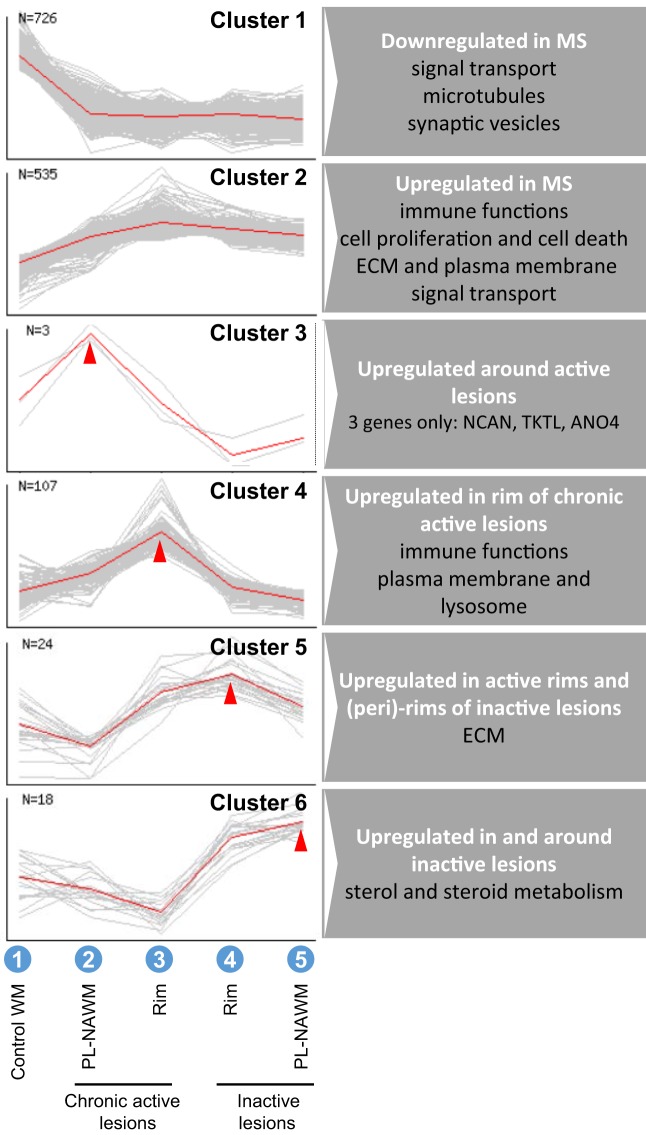
Cluster analysis of gene expression in and around multiple sclerosis (MS) lesions. Analysis was done with the sequence: (1) control white matter (WM), (2) chronic active peri-lesional (PL)-normal appearing white matter (NAWM), (3) chronic active rim, (4) inactive rim, (5) inactive PL-NAWM (also shown in Figure 1), which resulted in six different expression patterns, representing overall differences between control and MS (clusters 1 and 2), specific upregulation around expanding chronic active lesions (cluster 3), specific upregulation in active rims (cluster 4), upregulation in active rims and (peri)-rims of inactive lesions (cluster 5), or upregulation in and around inactive lesions (cluster 6). *N* indicates the number of significantly regulated genes within a cluster.

A cluster analysis was performed using the NIA Microarray Analysis Software to visualize gene expression during the presumed, subsequent stages of MS lesion activity and halt. The following sequence was chosen as input: (1) control WM, (2) chronic active PL-NAWM, where initial events in demyelination may be present in case the chronic active lesion was expanding, (3) chronic active rim, where active demyelination is ongoing, (4) inactive rim, where earlier demyelination has ceased, and (5) inactive PL-NAWM, where lesion progression has stopped (Figure 1; Arabic numbers). This gene cluster analysis revealed six specific patterns of gene expression between the subgroups tested (Figure 3). Some genes were generally expressed lower (cluster 1) or higher (cluster 2) in all lesion subregions, compared to control tissue. The other four patterns followed the presumed sequence of MS lesion development with a peak of gene expression around chronic active lesions (cluster 3), a peak of gene expression in the rim of chronic active lesions (cluster 4), low gene activity around active rims, but high expression in active rims and (peri)-rims of inactive lesions (cluster 5), and high gene expression in and around inactive lesions, but low activity in active rims (cluster 6).

The GO of the specific gene expression clusters was analyzed by GOstat (19) (Table S4 in Supplementary Material). The GO shows which gene functions are overrepresented within the groups studied, compared to all genes measured on the microarray. The most robust associations (i.e., with many GO terms changed) were found in cluster 2 and 4. In cluster 2, with overall high expression in MS, genes involved in immune functions were overrepresented, which is in line with MS being an inflammatory disease. In cluster 4, with highest expression in the active rim, genes involved in immune response/antigen presentation and cellular compartmentation, e.g., membrane and lysosome, were overrepresented, corresponding with the process of demyelination. In cluster 1, cluster 5, and cluster 6, associations were less robust. In cluster 1, with overall low expression in MS, various cellular functions linked with homeostasis were overrepresented. In cluster 5, with high expression in active rims and (peri)-rims of inactive lesions, but low activity in peri-rims of chronic active lesions, genes involved in extracellular matrix and/or collagen synthesis were overrepresented. In cluster 6, with high expression in and around inactive lesions, but low activity in active rims, sterol biosynthesis was overrepresented. The number of genes that fell within cluster 3 (*n* = 3) was too low for the GO analysis. The GO also showed a shift in the expression location of overrepresented genes from the plasma membrane to the lysosomal membrane from comparison II to comparison I (data not shown). Furthermore, processes involved in lipid metabolism were overrepresented in comparison I (data not shown).

### Selection of Genes of Interest

Genes of interest were selected based on their regulation between the subgroups (direct comparisons) and their expression pattern in the cluster analysis, with a specific interest of genes regulated peri-lesionally around chronic active lesions.

*CHIT1* (chitinase 1) *GPNMB* (glycoprotein non-metastatic melanoma protein B), and *CCL18* (C–C motif chemokine ligand 18) were the most upregulated genes in comparison I. *CHIT1* showed the highest expression in the rim of chronic active MS lesions, with a 10.2-fold change, compared to the expression in the rim of inactive MS lesions (Figure 2). *CHIT1* was also slightly upregulated in the PL-NAWM of chronic active MS lesions, compared to the PL-NAWM of inactive MS lesions (fold change of 2.0). *GPNMB* and *CCL18* showed a peak in expression in the rim of chronic active MS lesions, compared to the rim of inactive MS lesions, with a fold change of 8.0 and 6.8, respectively (Figure 2). Moreover, *GPNMB* was most highly induced around chronic active lesions compared to control tissue (fold change of 7.1) in comparison III.

Myelin recognition and uptake requires the presence of dedicated receptors at the surface of microglia/macrophages. We previously reported selective upregulation of scavenger receptors in and around demyelinating areas in MS (14). Congruently, genes upregulated in the rim of chronic active MS lesions compared to the rim of inactive MS lesions (comparison I) included the scavenger receptors *OLR1* [oxidized low-density lipoprotein receptor 1, also known as lectin-type oxidized LDL receptor 1 (LOX-1)], *CD68* (cluster of differentiation 68), *MSR1* [macrophage scavenger receptor 1, also known as scavenger receptor AI/II (SR-AI/II)], and *CXCL16* (C-X-C motif chemokine ligand 16) (fold change of 2.8–1.8) (Figure 2). *OLR1, CD68*, and *MSR1* were also upregulated in the PL-NAWM of chronic active MS lesions, compared to the PL-NAWM of inactive MS lesions and to control tissue (comparisons II and III), indicating that these molecules may be involved in initial demyelination. All scavenger receptors showed the highest expression in the rim of chronic active MS lesions.

Three other molecules of interest were *CXCR4* (C-X-C motif chemokine receptor 4), *NPY* (neuropeptide Y), and *KANK4* (KN motif and ankyrin repeat domains 4). The chemokine receptor *CXCR4* was upregulated in comparison I (fold change of 4.1) (Figure 2). The neurotransmitter *NPY* had the highest fold change in comparison II (fold change of 5.9) (Figure 2). Expression was highest in control tissue and PL-NAWM of chronic active MS lesions, lower in the rim of chronic active MS lesions, and lowest in inactive MS lesions (data not shown). The cytoplasmic protein *KANK4* had the highest *p*-value and was downregulated in the PL-NAWM of chronic active MS lesions compared to the PL-NAWM inactive lesions (comparison II; fold change of 0.3) (Figure 2). Expression was highest in inactive MS lesions and lower in control tissue and in chronic active MS lesions (data not shown).

Finally, *NCAN* (neurocan), *TKTL1* (transketolase-like 1), and *ANO4* (anoctamin 4) were included because these were the only genes in cluster 3, with a specific peak in expression in the PL-NAWM of chronic active MS lesions (Figure 3). *NCAN* and *ANO4* are also upregulated in the PL-NAWM of chronic active MS lesions compared to the PL-NAWM of inactive lesions (comparison II; fold change of 2.7 and 2.1) (Figure 2). Immunohistochemical staining showed more explicit ANO4 expression in the PL-NAWM of chronic active lesion compared to PL-NAWM of inactive MS lesion (Figure S2 in Supplementary Material).

### Validation and Further Characterization of Genes of Interest

There was a significant difference of the RIN value of control tissue compared to all MS lesion subareas, which is not unexpected as the control tissue was dissected in the cryostat using a scalpel as opposed to laser-based microdissection of the MS tissue. This significant difference did not influence our conclusions, as microarray data were validated by qPCR (Table 2), which showed no effect of RIN value on expression levels when normalizing with housekeeping genes (20). There was no significant difference in RIN values between the different MS lesion areas.

**Table 2:** Selected genes of interest.

Gene symbol	Comparison I: chronic active rim vs inactive rim				Comparison II: chronic active PL-NAWM vs inactive PL-NAWM				Comparison III: chronic active PL-NAWM vs control			
	Microarray		qPCR		Microarray		qPCR		Microarray		qPCR	
	*p*-Value	Fold change	*p*-Value	Fold change	*p*-Value	Fold change	*p*-Value	Fold change	*p*-Value	Fold change	*p*-Value	Fold change
CHIT1	1.07E−18	10.2	7.00E−03	36.0	4.28E−02	2.0	n.d.	n.d.	n.s.	n.a.	1.20E−03	5.8
GPNMB	3.61E−08	8.0	3.00E−04	12.4	n.s.	n.a.	2.20E−03	8.3	7.40E−06	7.1	3.10E−03	6.7
CCL18	2.13E−15	6.8	4.76E−02	66.7	n.s.	n.a.	n.s.	n.a.	n.s.	n.a.	n.s.	n.a.
OLR1	2.76E−04	2.9	5.90E−03	3.2	4.36E−02	2.1	6.00E−04	7.5	4.86E−06	4.2	2.00E−04	6.0
CD68	1.55E−07	2.8	6.00E−04	5.2	3.15E−02	1.7	3.00E−04	4.8	3.16E−02	2.2	4.00E−04	5.0
MSR1	2.09E−04	2.8	5.90E−03	4.0	4.18E−02	1.8	2.05E−02	5.3	1.64E−04	4.8	7.00E−04	7.3
CXCL16	4.99E−04	1.8	1.75E−02	2.9	n.s.	n.a.	9.30E−03	2.8	n.s.	n.a.	n.s.	n.a.
CXCR4	1.08E−04	4.1	n.s.	n.a.	n.s.	n.a.	n.s.	n.a.	n.s.	n.a.	n.s.	n.a.
NPY	n.s.	n.a.	4.01E−02	6.0	1.15E−03	5.9	4.10E−03	22.9	n.s.	n.a.	n.s.	n.a.
KANK4	7.36E−09	0.4	1.40E−02	0.2	5.65E−06	0.3	n.s.	n.a.	n.s.	n.a.	6.80E−03	0.2

*n.a., not applicable; n.d., not determined; n.s., not significant; PL-NAWM, peri-lesional normal appearing white matter; qPCR, quantitative polymerase chain reaction*.

Significant differences in gene expression were confirmed for *CHIT1, GPNMB, CCL18, KANK4, OLR1, CD68, MSR1*, and *CXCL16* in comparison I; for *NPY, OLR1, CD68*, and *MSR1* in comparison II; and for *GPNMB, OLR1, CD68*, and *MSR1* in comparison III. Some genes that showed no significant difference with microarray, did show a significant different with qPCR, e.g., *NPY* in comparison I, *GPNMB* and *CXCL16* in comparison II, and *CHIT1* and *KANK4* in comparison III. *CXCR4* showed no significant difference when validated with qPCR.

The expression pattern for most genes was similar with qPCR as compared to the microarray. *CHIT1, GPNMB, CCL18, CXCR4, OLR1, CD68, MSR1*, and *CXCL16* all showed highest expression in the rim of chronic active MS lesions. *GPNMB, OLR1, CD68*, and *MSR1* were upregulated in comparison II and III, and *CXCL16* in comparison II. Expression of *NPY* was highest in control tissue and the PL-NAWM of chronic active MS lesions, lower in the rim of chronic active MS lesions, and lowest in inactive MS lesions. The expression pattern for *KANK4* was different with qPCR. Expression levels for *KANK4* peaked in inactive MS lesions with microarray, but showed a peak in control tissue with qPCR. Within MS subregions, *KANK4* expression was still highest in the rim of inactive MS lesions.

**Figure 4:**
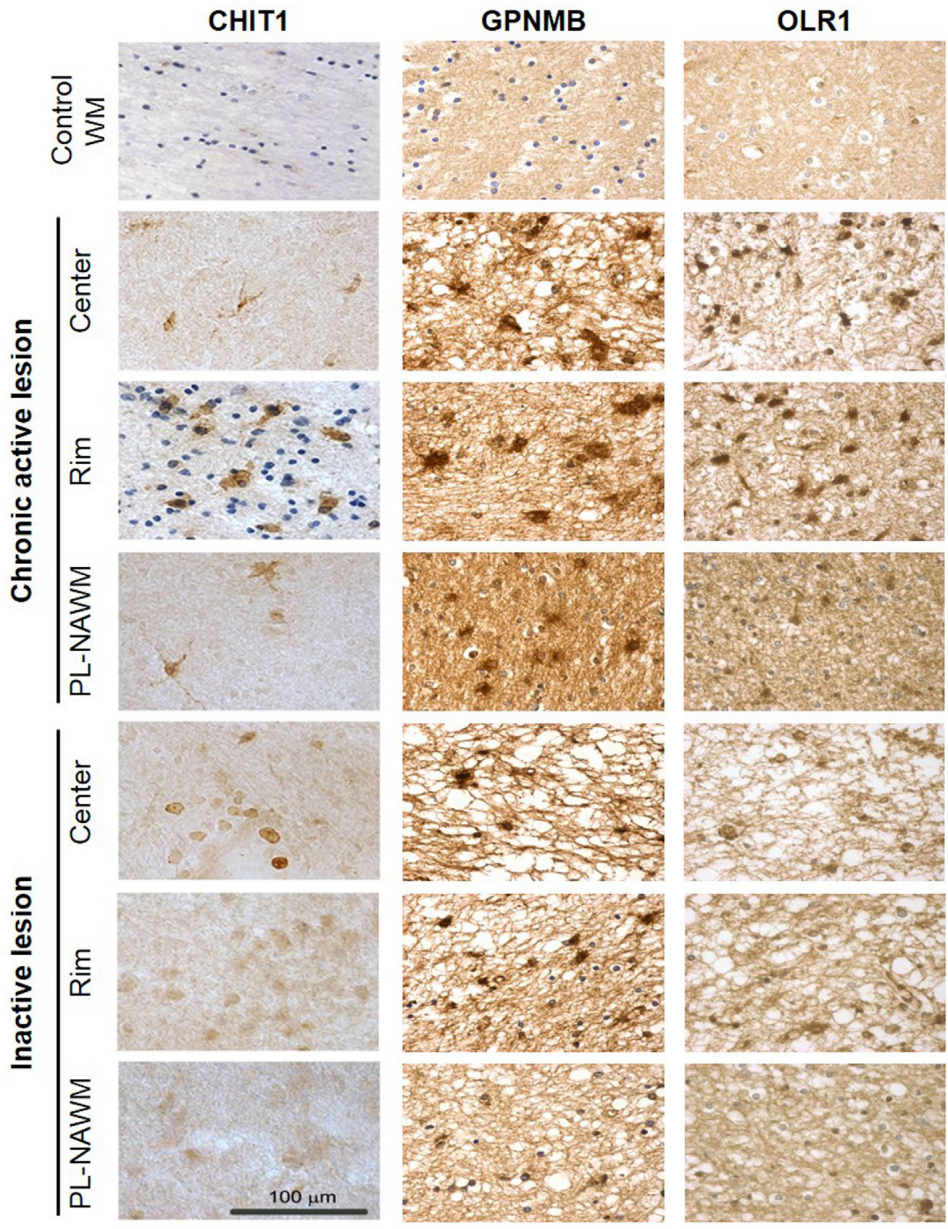
Expression of CHIT1, GPNMB, and OLR1 in and around multiple sclerosis (MS) lesions. Protein expression of CHIT1, GPNMB, and OLR1 in control tissue and in the center, rim, and peri-lesional (PL)-normal appearing white matter (NAWM) of chronic active and inactive MS lesions determined by immunohistochemistry. Scale bar = 100 µm.

Expression of *CHIT1, GPNMB*, and *OLR1* was further investigated at the protein level by immunohistochemistry (Figure 4). CHIT1, GPNMB, and OLR1 were not detected in control tissue, but were clearly present in chronic active MS lesions in the gliotic center, the rim, and also the peri-rim, following the RNA expression pattern. In inactive MS lesions, only GPNMB expression was identified in the center of the lesion and the lesion rim. Protein expression of CD68, MSR1, and CXCL16 in and around chronic active MS lesions has been described previously by us (14).

**Figure 5:**
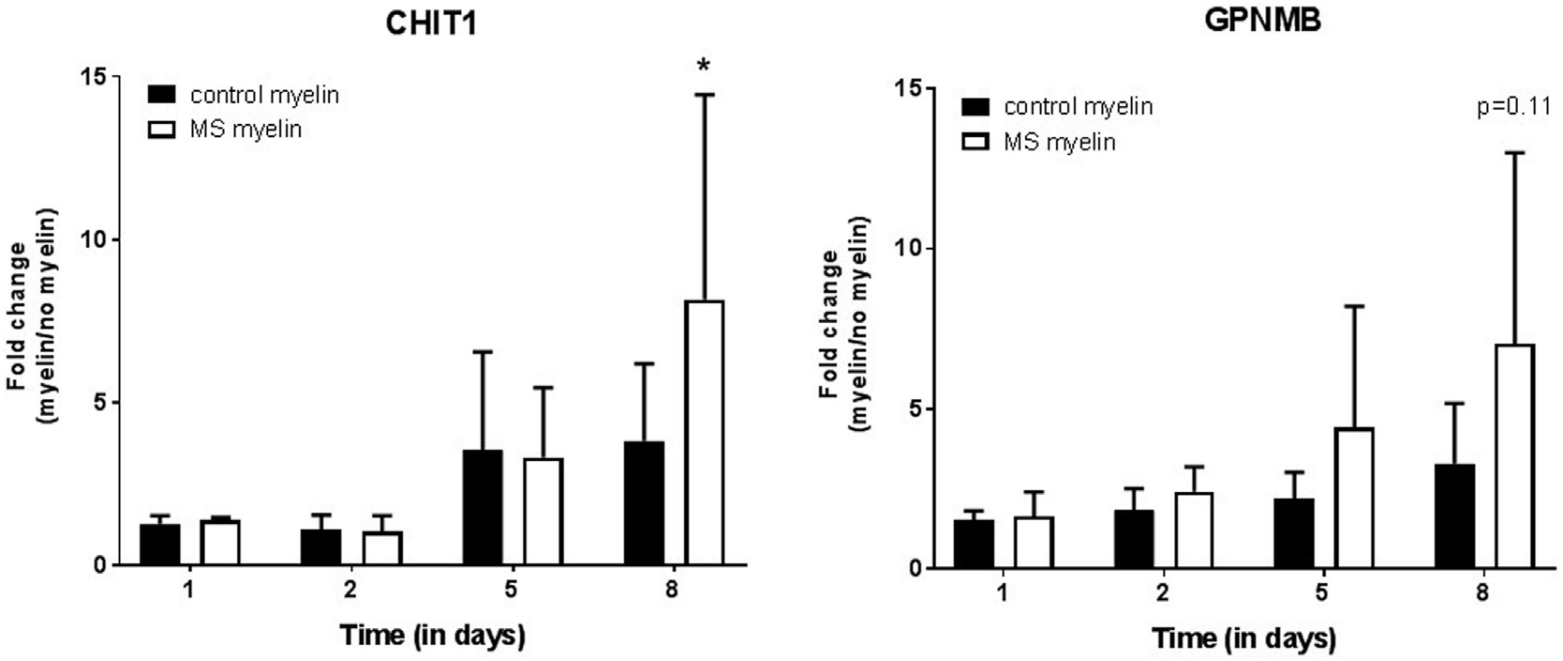
Upregulation of *CHIT1* after uptake of multiple sclerosis (MS) myelin *in vitro*. At the mRNA level, both *CHIT1* (*p* = 0.02) and *GPNMB* (*p* = 0.007) are upregulated in THP-1 macrophages after incubation with myelin from MS donors for 5 days, followed by 3 days incubation in normal medium. Fold change from macrophages cultured without myelin, *n* = 3, *p < 0.05.

Notably, these three genes have also been linked with Gaucher disease, a lysosomal storage disorder (see Discussion) and may relate to the process of myelin ingestion by microglia/macrophages. We next tested whether expression of *CHIT1* and *GPNMB* is increased upon myelin uptake, as this has been demonstrated for *CCL18* (21). Differentiated THP-1 macrophages were exposed to myelin from MS or control donors for 5 days, followed by 3 days culture in normal medium (Figure 5). After 8 days in culture, cells cultured with MS myelin showed a significant increase in *CHIT1* expression (*p* = 0.02) and a trend toward more *GPNMB* (*p* = 0.07) expression, compared to no myelin uptake. Uptake of myelin from control donors and myelin ingestion over time did not show an altered expression pattern. To conclude, both *CHIT1* and *GPNMB* are highly expressed in the rim and peri-rim of chronic active lesions, likely due to the uptake of MS myelin by microglia/macrophages.

### The Effect of Scavenger Receptor Knockdown on Myelin Phagocytosis *In Vitro*

**Figure 6:**
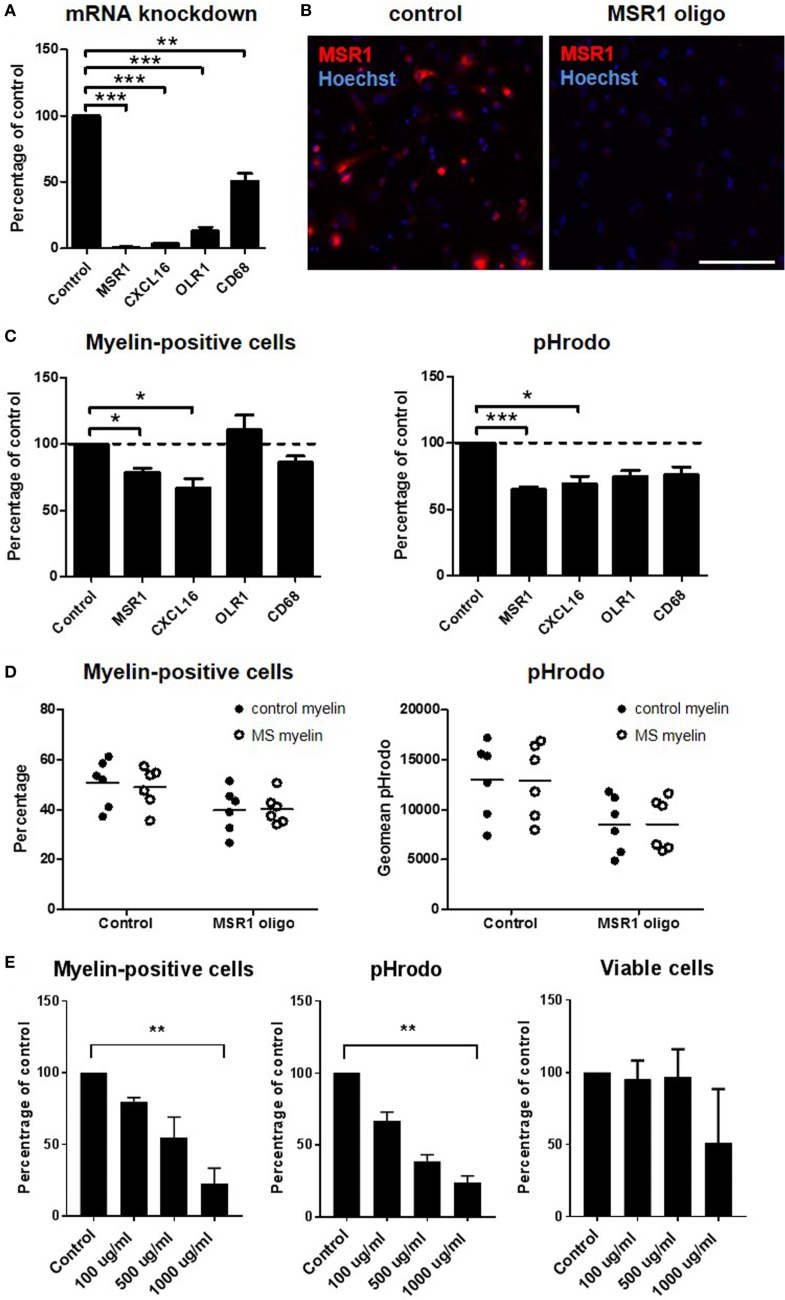
Functional role of scavenger receptors in myelin phagocytosis *in vitro*. *MSR1, CXCL16, OLR1*, and *CD68* were downregulated with antisense oligonucleotides in the human macrophage cell line THP-1. Silencing efficiency was determined on mRNA level with quantitative polymerase chain reaction **(A)** and on protein level with immunocytochemistry [**(B)**; only shown for MSR1]. Uptake of pHrodo-labeled myelin was validated by flow cytometry **(C)** and compared for myelin obtained from control and multiple sclerosis (MS) brain tissue **(D)**. The number of cells that had phagocytosed myelin, and the total amount of myelin phagocytosed (geomean pHrodo) were calculated. The number of independent experiments (*n*) was 6 (MSR1), 4 (CXCL16), 3 (OLR1), and 3 (CD68). **(E)** Fucoidan was used to inhibit a broad spectrum of scavenger receptors in THP-1 cells. Provided is the number of cells that had phagocytosed myelin, the total amount of myelin phagocytosed (geomean pHrodo), and the viability of cells at the time point of analysis (*n* = 3). Scale bar in panel **(B)** = 200 µm *p < 0.05, **p < 0.01, ***p < 0.005.

As this study and our earlier work (14) revealed high expression of scavenger receptors in the rim as well as around chronic active MS lesions, we studied the role of *OLR1, CD68, MSR1*, and *CXCL16* in an *in vitro* myelin phagocytosis assay (Figure 6). Antisense oligonucleotides were developed that downregulate expression of these genes in the human macrophage cell line THP-1, confirmed by qPCR (Figure 6A). Immunocytochemistry confirmed the efficient knockdown of MSR1 (Figure 6B). Knockdown of *MSR1* and *CXCL16* significantly decreased the percentage of macrophages that phagocytosed myelin and the total myelin phagocytosed, compared to untreated cells (Figure 6C). Phagocytosis of myelin derived from control or MS tissue was similarly reduced by *MSR1* knockdown (Figure 6D). To block scavenger receptor function in a redundant manner, we also applied a broad pharmacological inhibitor (22, 23). Fucoidan showed a non-toxic, dose-dependent significant reduction of the percentage of macrophages that phagocytosed myelin and the total amount of myelin phagocytosed at 1,000 µg/ml (*p* = 0.007; Figure 6E). Upregulation of scavenger receptors in chronic active MS lesions thus likely contributes to demyelination.

## Discussion

In this study, we compared gene expression in and around chronic active MS lesions, inactive MS lesions, and control tissue to identify gene expression related to lesion activity and lesion halt. We found upregulation of genes involved in immune function, lipid binding, and lipid uptake in the active rim. This confirms the expectation, since in rims of chronic active lesions, inflammatory microglia/macrophage phagocytose myelin. Importantly, around chronic active MS lesions, genes involved in lipid binding and uptake also showed increased expression. This indicates early demyelination around chronic active lesions, showing that these lesions are indeed expanding. In addition, genes with a possible anti-inflammatory and/or neuroprotective function were upregulated in rims and around chronic active expanding lesions, possibly relating to the induction of endogenous protective mechanisms. Based on direct comparisons and cluster analysis, and with a specific focus on genes related to lesion activity and expansion, we identified several genes of interest: *CHIT1, GPNMB, CCL18, OLR1, CD68, MSR1, CXCL16, CXCR4, NPY, KANK4, NCAN, TKTL1*, and *ANO4*.

### Altered Gene Expression in the Rim and PL-NAWM of Chronic Active and Inactive MS Lesions

To visualize gene expression during MS lesion progression, we used the set-up as shown in Figure 1, which was thought to best resemble the sequence of events in MS lesion formation and progression. Cluster analysis (Figure 3) revealed six different expression patterns representing overall differences between control and MS (clusters 1 and 2), specific upregulation only around expanding, chronic active lesions (cluster 3), specific upregulation in active rims (cluster 4), upregulation in active rims and around inactive lesions, but low activity around active rims (cluster 5), or upregulation in and around inactive lesions, but low activity in active rims (cluster 6). Overrepresented genes within each cluster were detected by GO analysis (Table S4 in Supplementary Material). Clusters 2 and 4 showed genes involved in immune functions, which is expected as these genes peak in the rim of chronic active MS lesions. Not unexpected, genes involved in the lysosomal activity were overrepresented in cluster 4, corresponding with the process of demyelination. Genes involved in sterol and steroid metabolism were overrepresented in cluster 6, possibly indicating an attempt to repair damaged axons and myelin after active demyelination has diminished. In cluster 3, only three genes were regulated (discussed below).

The GO analysis also showed a shift in the expression location of overrepresented genes from the plasma membrane to the lysosomal membrane from comparison II to comparison I (data not shown). This is not surprising, as myelin first needs to be recognized and phagocytosed, before it can be processed in the lysosomes. Furthermore, processes involved in lipid metabolism were overrepresented in comparison I (data not shown), indicating that phagocytosed myelin is being processed.

### Joint Upregulation of CHIT1, GPNMB, and CCL18 Is Mediated by Myelin Uptake

Our study identified *CHIT1, GPNMB*, and *CCL18* as top-3 upregulated genes in the rim of chronic active MS lesions, where foamy, myelin-accumulating microglia/macrophages are abundant. Interestingly, also in regions surrounding chronic active lesions, *CHIT1* and *GPNMB* were upregulated. Enhanced expression of *CCL18* in myelin-laden macrophages *in vitro* and in the rim and center of active MS lesions has been reported before (21). Here, we demonstrate a relation between the regulation of both, *CHIT1* and *GPNMB*, and myelin uptake. Both genes showed an increased expression in THP-1-derived macrophages after 8 days of incubation with MS myelin, and not with control myelin, suggesting that specifically myelin derived from MS donors induce *CHIT1* and *GPNMB* expression. Wheeler and colleagues already reported that the composition of MS myelin in NAWM is altered, compared to control myelin (24), and previously, we described that MS myelin is taken up more efficiently (18).

Of note, upregulation of *CHIT1* and *CCL18* in lipid-laden macrophages of Gaucher patients has long been known (25, 26). More recently, *CHIT1* has been described as a prognostic biomarker for early MS (27, 28), and soluble *GPNMB*, which is secreted from different cell types through a disintegrin and metalloproteinase 10 sheddase activity, was identified as a further biomarker of Gaucher disease (29, 30). Co-regulation of *CHIT1, GPNMB*, and *CCL18* strengthens the idea that lipid-accumulating Gaucher cells and myelin-phagocytosing microglia/macrophages share cellular characteristics (21), possibly related to lysosomal stress, a function overrepresented in active rims (cluster 4). The precise relationship between the induction of *CHIT1, GPNMB*, and *CCL18*, myelin processing, and lysosomal activities of microglia in MS warrants further investigation.

Functional studies have linked *CHIT1, GPNMB*, and *CCL18* primarily with the suppression of inflammation. Inhibition of *CHIT1* in a mouse macrophage cell line induced a pro-inflammatory phenotype *in vitro*, caused downregulation of *MSR1* and CD68, and decreased cholesterol uptake (31). Notably, *CHIT1* levels in the CSF reliably indicate microglial activation in clinical trials (32). *GPNMB* was upregulated in astrocytes and neurons in an animal model of amyotrophic lateral sclerosis, and secretion of the extracellular fragment by astrocytes had a neuroprotective effect (33). Another study showed that *GPNMB* was mainly expressed by macrophages/microglia in the rat brain and was upregulated in inflammatory conditions (34), acting as a negative regulator to prevent excessive immune responses (35). In line herewith, *Gpnmb*-deficient mice (DC-HIL^−/−^) manifested exacerbated autoimmune encephalomyelitis (EAE) (36). Finally, *CCL18* recruited a subset of human regulatory T cells *in vitro*, which suppressed proliferation of effector T cells via interleukin-10 production (37). *CCL18* is also produced by macrophages that have ingested myelin and show an immunosuppressive phenotype (21). Suppressive effects on inflammation by *CHIT1, GPNMB*, and *CXCL18* in relation to demyelination in regions surrounding active MS lesions need to be elucidated.

### Scavenger Receptors Upregulated in and around Chronic Active MS Lesions Mediate Myelin Uptake

Myelin uptake during demyelination likely depends on scavenger receptors. We found *OLR1, CD68, MSR1*, and *CXCL16* upregulated in all three comparisons (*CXCL16* only in comparison I and in comparison II with qPCR). Thus, these scavenger receptors are upregulated in chronic active rims, but also around chronic active lesions, indicating that they are involved in early demyelination. We cannot fully exclude some contamination of PL regions with rim tissue during laser dissection, but early demyelination around chronic active lesions is further indicated by immunohistochemistry, showing upregulation of *CHIT1, GPNMB*, and *OLR1* in and around chronic active MS lesions. These results extends our earlier work, showing enhanced expression of *CD68, MSR1*, and *CXCL16* in and around chronic active MS lesions, compared to control tissue (14). *CD68* is a scavenger receptor predominantly expressed on the lysosomal membrane, but the small percentage expressed on the plasma membrane is capable of oxLDL phagocytosis (38, 39). SA-RI/II, encoded by *MSR1*, was shown to be directly involved in myelin uptake *in vitro* (40–42), and *Msr1*^−/−^ mice showed less severe disease and reduced demyelination in the EAE model (43). *CXCL16* has a dual function as a scavenger receptor and as a chemokine in soluble form. Neutralizing antibodies against *CXCL16* delayed the onset and reduced the severity of EAE in mice (44). The soluble form is elevated in MS patients, compared to control subjects (45). This indicates that scavenger receptors could be actively involved in demyelination in MS. Furthermore, their upregulation in the PL-NAWM of chronic active MS lesions, might suggest they are involved in the initial stages of MS lesion development and progression.

The functional role of *OLR1, CD68, MSR1*, and *CXCL16* was studied in an *in vitro* myelin phagocytosis assay, using the human macrophage cell line THP-1. All genes were significantly downregulated, compared to untreated cells. Downregulation of *MSR1* and *CXCL16* resulted in a significant decrease in the number of myelin-phagocytosing cells and total myelin uptake. A direct role of *MSR1* in myelin phagocytosis in MS is consistent with earlier research (40–42) and supported by its expression in regions of active demyelination in MS brain tissue. Its upregulation in the PL-NAWM of chronic active MS lesions could indicate that phagocytosing cells in yet unaffected areas are already preparing for demyelination. Notably, pharmacological inhibition of a broad spectrum of scavenger receptors by fucoidan further reduced the number of phagocytosing macrophages and the total amount of myelin uptake, indicating that a combination of scavenger receptors contributes to the uptake of myelin.

### Molecules Associated with Lesion Expansion

Our analysis identified many more genes regulated in and around chronic active and inactive MS lesions, which are potential targets to regulate lesion expansion. *NCAN, TKTL1*, and *ANO4* were of specific interest because they peaked at the PL-NAWM of chronic active MS lesions (cluster 3). Consistent with our findings, an upregulation of the extracellular matrix component *NCAN* in the rim and also slightly in the PL-NAWM of MS lesions has been reported earlier (46). Furthermore, *NCAN* is expressed by astrocytes and known to be upregulated after brain injury and modulates neuronal outgrowth (47). *TKTL1* is a transketolase expressed by mature oligodendrocytes in PL-NAWM of MS lesions and by oligodendrocyte precursors, reactive astrocytes, and macrophages in the rim of MS lesions (48) that has been postulated to prevent neurodegeneration by reducing the formation of advanced glycation end products and radicals (49). Upregulation of *TKTL1* might be an initial protective reaction to changes taking place in the PL-NAWM of MS lesions. Both *NCAN* and *TKTL1* might be important regulators in early axonal damage. Finally, the potential role of *ANO4*, a suggested Ca^2+^-dependent lipid scramblase, in MS lesion development is unknown. In contrast to chronic active lesions, the PL-NAWM of inactive lesions show overrepresented genes involved in sterol biosynthesis, which might indicate an attempt to restore damaged tissue and myelin after active demyelination has diminished.

Both *KANK4* and *NPY* are regulated in the RIM and PL-NAWM of chronic active lesion, where *KANK4* was downregulated and *NPY* showed an upregulation. KANK family proteins are involved in the inhibition of actin stress fibers formation and cell motility [reviewed in Ref. (50)], but the exact function of *KANK4* and its role in MS lesions formation needs to be determined. In contrast to *KANK4*, the neurotransmitter *NPY* was upregulated in chronic active lesions. Application of *NPY* during EAE induction significantly suppressed clinical signs in DA rats (51) and in mice (52). *NPY* also inhibited disease and reduced inflammation when administered after the onset of EAE symptoms (53). Fc receptor-dependent phagocytosis of opsonized latex beads by lipopolysaccharide-stimulated microglia was reduced by *NPY* (54). Induction of *NPY* in and around acute MS lesions, might regulate myelin phagocytosis and immune suppression during early demyelination events.

Studying gene expression profiles associated with expansion of chronic active MS lesions is essential, since we recently showed that chronic active lesions highly correlate to disease progression (Luchetti et al., submitted). Taken together, we found changes in immune activation and lipid uptake in the rim of chronic active MS lesions. Genes related to lipid phagocytosis were also upregulated in the PL-NAWM of chronic active MS lesions, showing that chronic active lesions indeed expand. Importantly, potentially protective, anti-inflammatory genes were also upregulated in the (peri)-rim of chronic active MS lesions, suggesting a vain attempt to prevent lesion expansion and progression. We functionally confirmed the ability of the scavenger receptors *MSR1* and *CXCL16* to mediate myelin uptake. Our results pinpoint scavenger receptors as interesting targets to stop demyelination around chronic active lesions, which could block lesion expansion.

## Ethics Statement

This study was carried out in accordance with the recommendations of the Netherlands Brain Bank (NBB, Amsterdam, The Netherlands). The NBB has obtained approval from the ethical medical committee of VU University medical center (VUmc, Amsterdam, The Netherlands) for all documents and procedures of brain banking, including the informed consent for the use of tissue and medical records for research purposes. Ethical, judicial, and anonymization strategies are all according to the published code of conduct for European brain banks (see for ethical approval, ethical documents and the ethical code of conduct for Brain banking: https://www.brainbank.nl/about-us/the-nbb/).

## Author Contributions

DH carried out tissue laser dissections, microarray hybridizations, gene expression analyses, immunohistochemical stainings and drafted the manuscript. JS performed immunohistochemical stainings and phagocytosis experiments. MP performed phagocytosis experiments and helped with drafting of the manuscript. KB carried out gene expression analyses. KS isolated microglia and myelin. CE performed qPCRs and immunohistochemical stainings. EH participated in study coordination and data analysis. JH and IH were responsible for study design and coordination, data interpretation, and drafting of the manuscript. All authors have read and approved the final manuscript.

## Conflict of Interest Statement

The authors declare that the research was conducted in the absence of any commercial or financial relationships that could be construed as a potential conflict of interest.

## Data Availability

Microarray data have been uploaded in the Gene Expression Omnibus (GEO) database. The GEO accession number to access the complete dataset is: GSE108000.

## Footnotes

^1^http://www.brainbank.nl.

^2^http://www.bioconductor.org.
