# Supplementary material for: Gene Expression Profiling of Multiple Sclerosis Pathology Identifies Early Patterns of Demyelination Surrounding Chronic Active Lesions
Source: Front Immunol. 2017 Dec 21;8:1810. doi: 10.3389/fimmu.2017.01810 (PMC5742619; doi:10.3389/fimmu.2017.01810)
Supplement: Supplementary file 1 [file Image_1.PDF]

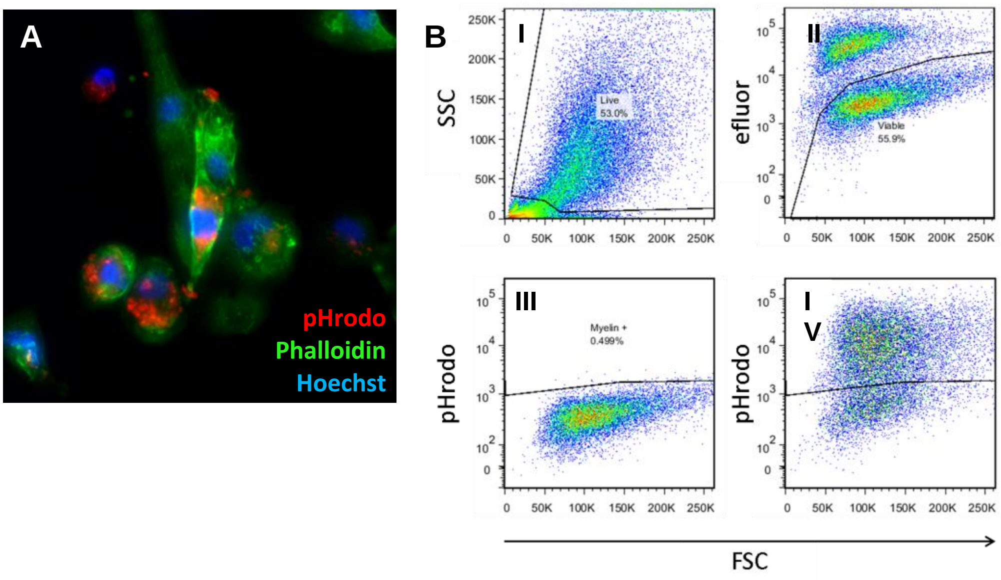

Supplemental Figure 1. Myelin phagocytosis by the human macrophage cell line THP-1. (**A**) pHrodo-labeled myelin emits a red fluorescent signal upon phagocytosis. For immunocytochemistry, Hoechst was used to stain the nucleus, and phalloidin was used to stain the actin and visualize the cell morphology. (**B**) Histograms showing myelin uptake (pHrodo-positivity) in the viable cell population (eFluor-negative) analyzed by flow cytometry. Debris was excluded based on particle size in panel I. Panel I-III: sample without myelin added; panel IV: sample with pHrodo-labelled myelin. FSC = forward scatter; SSC = side scatter. Scale bar in A = 50 µm
