# Supplementary material for: Gene Expression Profiling of Multiple Sclerosis Pathology Identifies Early Patterns of Demyelination Surrounding Chronic Active Lesions
Source: Front Immunol. 2017 Dec 21;8:1810. doi: 10.3389/fimmu.2017.01810 (PMC5742619; doi:10.3389/fimmu.2017.01810)
Supplement: Supplementary file 2 [file Image_2.PDF]

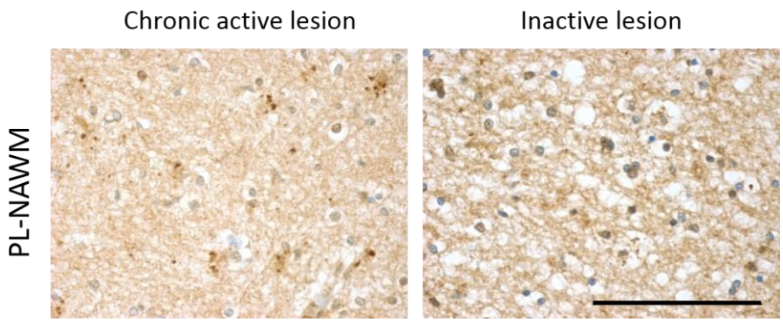

Supplemental Figure 2. Expression of ANO4 at the perilesional site of MS lesions. Protein expression of ANO4 is determined by immunohistochemistry and shows a punctate staining pattern around chronic active MS lesion. Scale bar = 100 μm
