# Supplementary material for: Gene Expression Profiling of Multiple Sclerosis Pathology Identifies Early Patterns of Demyelination Surrounding Chronic Active Lesions
Source: Front Immunol. 2017 Dec 21;8:1810. doi: 10.3389/fimmu.2017.01810 (PMC5742619; doi:10.3389/fimmu.2017.01810)
Supplement: Supplementary file 3 [file Table_1.PDF]

Supplemental Table 1. Donor characteristics

NBB no.	Tissue	Age	Sex	Type	Duration	Cause of death	PMD	pH	IHC
1995-043	C	75	M			Sepsis, adenocarcinoma	07:20	6.16	
1996-014	C	54	F			Renal failure	08:00	6.45	
1996-032	C	60	F			Legal euthanasia	08:25	6.6	
1996-129	C	70	M			Pancreas carcinoma	07:30	6.4	
1997-042	C	65	F			Cardiac arrest	12:50	6.94	
2000-025	C	41	F			Bleeding from adenocarcinoma	13:30	6.23	
2000-050	C	52	F			Metastasized leiomyosarcoma	06:50	7.16	
2001-069	C	68	F			Legal euthanasia	07:37	5.45	+
2008-073	C	50	F			Metastasized bronchocarcinoma	04:10	6.98	
2009-003	C	62	M			Unknown	07:20	6.36	+
1996-121	CA	53	F	RR	18	Pneumonia	07:16	6.54	
1997-160	CA	40	F	RR	11	Pneumonia	07:00	6.33	
1999-051	CA	45	F	RR	14	Legal euthanasia	10:55	6.62	+
2001-018	CA	48	F	RR	9	Legal euthanasia	08:10	6.55	
2001-135	CA	43	M	RR	17	Pneumonia	08:30	6.48	+
2007-085	CA	66	F	RR	23	Unknown	06:00	6.18	+
2010-025	CA	51	M	SP	>20	Unknown	11:00	6.23	+
2001-093	I	66	F	RR	43	Liver failure	06:20	6.44	
2002-023	I	75	F	RR	34	Pneumonia	08:00	6.5	
2002-063	I	72	F	RR	14	Pneumonia	12:00	6.85	+
2008-053	I	64	F	RR	39	Urosepsis	10:10	6.3	
2008-100	I	77	F	RR	24	Legal euthanasia	10:00	6.5	+
2009-034	I	45	M	RR	19	Pulmonary embolism	07:45	6.22	+
2009-047	I	50	M	PP	24	Unknown	09:30	6.2	
2009-067	I	44	M	PP	13	Pneumonia	12:00	6.06	+
2010-040	I	57	F	U	25	Legal euthanasia	08:40	6.44	

Age = age at death (years); C = control subject; CA = chronic active MS; Duration = disease duration (years); F = female; I = inactive MS; IHC= immunohistochemistry; M = male; NBB no. = donor registration number of the Netherlands Brain Bank; pH = pH of CSF; PMD = post-mortem delay (h:min); PP = primary progressive MS; RR = relapsing-remitting MS; Tissue = tissue type; Type = clinical subtype of MS; U = unknown
