# Supplementary material for: Gene Expression Profiling of Multiple Sclerosis Pathology Identifies Early Patterns of Demyelination Surrounding Chronic Active Lesions
Source: Front Immunol. 2017 Dec 21;8:1810. doi: 10.3389/fimmu.2017.01810 (PMC5742619; doi:10.3389/fimmu.2017.01810)
Supplement: Supplementary file 4 [file Table_2.PDF]

Supplemental Table 2. Primer pairs used for qPCR

Gene symbol	Forward sequence (5'–3')	Reverse sequence (5'–3')
**CHIT1**	TGCGCAAATACAGCTTTGAC	ACCTCGTATCCAGCATCCAC
**GPNMB**	GCTGACTGTGAGACGAACCT	ACAGAAATCAGGGTGCTCGT
**CCL18**	CCCAGCTCACTCTGACCACT	GTGGAATCTGCCAGGAGGTA
**CXCR4**	TTCCCTCTAGTGGGCGGGG	AAAGGGCACTGAGACGCTGA
**NPY**	CATCACCAGGCAGAGATATGGA	ATCACCACATTGCAGGGTCT
**KANK4**	GCTCGCCAAGAACCTTCAAC	GGTCTTTCTCTTCATCCCCCT
**MSR1**	TTCAAAGCTGCACTGATTGCC	TTCTTCGTTTCCCACTTCAGGA
**CD68**	ACGCAGCACAGTGGACATTCT	TGATGCTCGAGTTGCTGCA
**CXCL16**	GCCATCGGTTCAGTTCATGA	AAAGGAGCTGGAACCTCGTGT
**OLR1**	TTACTCTCCATGGTGGTGCCT	TGCCCAGCACCATAATGGT
**GAPDH**	TGCACCACCAACTGCTTAGC	GGCATGGACTGTGGTCATGA
**TUBA1A**	CTTTGAGCCAGCCAACCAGA	GTACAACAGGCAGCAAGCCAT
**EEF1α**	AAGCTGGAAGATGGCCCTAAA	AAGCGACCCAAAGGTGGAT
