# Supplementary material for: Gene Expression Profiling of Multiple Sclerosis Pathology Identifies Early Patterns of Demyelination Surrounding Chronic Active Lesions
Source: Front Immunol. 2017 Dec 21;8:1810. doi: 10.3389/fimmu.2017.01810 (PMC5742619; doi:10.3389/fimmu.2017.01810)
Supplement: Supplementary file 5 [file Table_3.PDF]

Supplemental Table 3A. Top 50 upregulated genes in chronic active rim vs inactive rim (comparison I)

Gene symbol	Gene name	Adjusted *p*-value	Fold change
CHIT1	chitinase 1 (chitotriosidase)	1.07E-18	10.2
GPNMB	glycoprotein (transmembrane) nmb, transcript variant 1	3.61E-08	8.0
CCL18	chemokine (C-C motif) ligand 18 (pulmonary and activation-regulated)	2.13E-15	6.8
ACP5	acid phosphatase 5, tartrate resistant, transcript variant 4	9.86E-15	6.4
PKD2L1	polycystic kidney disease 2-like 1	8.91E-18	5.7
HLA-DQA1	major histocompatibility complex, class II, DQ alpha 1	3.19E-04	4.7
FCGR2B	Fc fragment of IgG, low affinity IIb, receptor (CD32), transcript variant 1	8.81E-11	4.6
CFD	complement factor D (adipsin)	2.48E-07	4.5
APOC1	apolipoprotein C-I	9.12E-07	4.4
CXCR4	chemokine (C-X-C motif) receptor 4, transcript variant 1	1.08E-04	4.1
PLA2G7	phospholipase A2, group VII (platelet-activating factor acetylhydrolase, plasma)	4.86E-10	4.0
HS3ST2	heparan sulfate (glucosamine) 3-O-sulfotransferase 2	4.68E-08	4.0
IFI30	interferon, gamma-inducible protein 30	4.54E-08	3.6
CAPG	capping protein (actin filament), gelsolin-like	1.24E-06	3.2
RAB42	RAB42, member RAS oncogene family	2.56E-07	3.0
MS4A7	membrane-spanning 4-domains, subfamily A, member 7, transcript variant 1	1.02E-05	3.0
ITGAX	integrin, alpha X (complement component 3 receptor 4 subunit)	5.41E-05	2.9
AMICA1	adhesion molecule, interacts with CXADR antigen 1, transcript variant 2	2.89E-09	2.9
DENND2D	DENN/MADD domain containing 2D	5.11E-08	2.9
OLR1	oxidized low density lipoprotein (lectin-like) receptor 1	2.76E-04	2.9
ALOX5AP	arachidonate 5-lipoxygenase-activating protein	1.51E-05	2.8
FGR	Gardner-Rasheed feline sarcoma viral (v-fgr) oncogene homolog, transcript variant 2	1.34E-05	2.8
CD68	CD68 molecule, transcript variant 1	1.55E-07	2.8
IRF5	interferon regulatory factor 5, transcript variant 1	4.33E-03	2.8
MSR1	macrophage scavenger receptor 1 (MSR1), transcript variant SR-AI	2.17E-04	2.8
ASCL2	achaete-scute complex homolog 2 (Drosophila)	8.53E-08	2.8
HLA-DMB	major histocompatibility complex, class II, DM beta	1.43E-03	2.8
SLC7A7	solute carrier family 7 (cationic amino acid transporter, y+ system), member 7, transcript variant 1	1.54E-06	2.8
TNFAIP2	tumor necrosis factor, alpha-induced protein 2	1.30E-06	2.7
BCL2A1	BCL2-related protein A1, transcript variant 1	3.17E-04	2.7
HLA-DRB5	major histocompatibility complex, class II, DR beta 5	2.04E-02	2.7
ADORA3	adenosine A3 receptor, transcript variant 1	5.84E-06	2.7
SLC47A1	solute carrier family 47, member 1	1.60E-13	2.7
MX2	myxovirus (influenza virus) resistance 2 (mouse)	2.13E-11	2.7
S100A11	S100 calcium binding protein A11	2.14E-06	2.6
NCF2	neutrophil cytosolic factor 2, transcript variant 1	1.63E-05	2.6
CD83	CD83 molecule, transcript variant 1	8.82E-05	2.6
FCGR2C	Fc fragment of IgG, low affinity IIc, receptor for (CD32)	2.13E-06	2.6
ENST00000424686	Major histocompatibility complex, class II, DQ beta 1	2.83E-03	2.6
HLA-DQA2	major histocompatibility complex, class II, DQ alpha 2	1.28E-04	2.6
SIGLEC8	sialic acid binding Ig-like lectin 8	4.72E-06	2.6
ENST00000412049	HLA class II histocompatibility antigen, DQ(2) alpha chain Precursor	3.34E-03	2.6
CCRL2	chemokine (C-C motif) receptor-like 2, transcript variant 1	5.68E-08	2.5
MS4A4A	membrane-spanning 4-domains, subfamily A, member 4, transcript variant 1	7.22E-03	2.5
FCER1G	Fc fragment of IgE, high affinity I, receptor for; gamma polypeptide	2.26E-06	2.5
HPSE	heparanase, transcript variant 1	2.04E-08	2.5
LAPTM5	lysosomal protein transmembrane 5	2.32E-03	2.5
LSP1	lymphocyte-specific protein 1, transcript variant 3	7.54E-03	2.5
HLA-DMA	major histocompatibility complex, class II, DM alpha	2.44E-04	2.5
KLHL6	kelch-like 6 (Drosophila)	8.32E-08	2.5

Supplemental Table 3B. Top 50 downregulated genes in chronic active rim vs inactive rim (comparison I)

Gene symbol	Gene name	Adjusted *p*-value	Fold change
HBB	hemoglobin, beta	1.44E-02	0.3
HBD	hemoglobin, delta	1.35E-02	0.3
CRLF1	cytokine receptor-like factor 1	2.86E-02	0.3
ABCA6	ATP-binding cassette, sub-family A (ABC1), member 6	1.20E-02	0.3
KANK4	KN motif and ankyrin repeat domains 4	4.88E-08	0.3
RNU2-2	RNA, U2 small nuclear 2, small nuclear RNA	1.30E-03	0.4
DHCR24	24-dehydrocholesterol reductase	1.93E-06	0.4
TSPAN8	tetraspanin 8	1.71E-05	0.4
ENST00000392423	titin isoform novex-3	4.30E-04	0.4
SQLE	squalene epoxidase	6.39E-06	0.5
ENST00000313339	Ankyrin repeat domain-containing protein 18A	3.75E-04	0.5
RBM11	RNA binding motif protein 11	6.49E-06	0.5
RNU1-5	RNA, U1 small nuclear 5, small nuclear RNA	1.15E-03	0.5
FSTL5	follistatin-like 5, transcript variant 1	2.35E-04	0.5
RMRP	RNA component of mitochondrial RNA processing endoribonuclease	5.46E-03	0.5
WIF1	WNT inhibitory factor 1 (WIF1)	3.37E-04	0.5
TTN	titin (TTN), transcript variant N2-A	4.19E-03	0.5
RELN	reelin, transcript variant 1	2.11E-03	0.5
LOC728449	Putative uncharacterized protein ENSP00000334090	8.82E-05	0.5
TNFRSF21	tumor necrosis factor receptor superfamily, member 21	8.82E-05	0.5
LOC100133402	hypothetical LOC100133402	6.36E-03	0.5
C21orf130	chromosome 21 open reading frame 130, non-coding RNA	4.18E-02	0.5
LOC100290344	FLJ44107 fis, clone TESTI4044296	3.57E-03	0.5
PDE11A	phosphodiesterase 11A, transcript variant 4	1.45E-04	0.5
SNORA28	small nucleolar RNA, H/ACA box 28, small nucleolar RNA	2.74E-03	0.5
ANKRD20B	ankyrin repeat domain 20B, non-coding RNA	1.71E-04	0.5
LDLR	low density lipoprotein receptor	1.06E-02	0.5
ALAS2	aminolevulinate, delta-, synthase 2, nuclear gene encoding mitochondrial protein, transcript variant 1	1.40E-02	0.5
KLK6	kallikrein-related peptidase 6, transcript variant B	1.97E-04	0.5
RAPGEF5	Rap guanine nucleotide exchange factor (GEF) 5	8.11E-05	0.5
MBP	myelin basic protein, transcript variant 7	6.71E-04	0.5
F5	coagulation factor V (proaccelerin, labile factor)	3.75E-04	0.5
HAPLN2	hyaluronan and proteoglycan link protein 2	1.09E-03	0.5
ABCA8	ATP-binding cassette, sub-family A (ABC1), member 8	1.68E-03	0.5
NIPAL4	NIPA-like domain containing 4	2.62E-04	0.5
CDKN1C	cyclin-dependent kinase inhibitor 1C (p57, Kip2), transcript variant 1	1.69E-05	0.5
GDF10	growth differentiation factor 10	4.17E-03	0.5
MOBP	myelin-associated oligodendrocyte basic protein	5.58E-03	0.5
HN1L	hematological and neurological expressed 1-like	1.76E-04	0.5
ENPP6	ectonucleotide pyrophosphatase/phosphodiesterase 6	5.81E-03	0.5
LOC283713	cDNA FLJ37663 fis, clone BRHIP2011120	3.80E-03	0.5
DPYSL5	dihydropyrimidinase-like 5	1.97E-04	0.5
INSIG1	insulin induced gene 1, transcript variant 1	1.83E-02	0.5
LOC100008587	5.8S ribosomal RNA (LOC100008587), ribosomal RNA	2.20E-02	0.6
TMEM125	transmembrane protein 125	4.85E-02	0.6
TMEM144	transmembrane protein 144	6.17E-03	0.6
SPOCK3	sparc/osteonectin, cwcv and kazal-like domains proteoglycan (testican) 3, transcript variant 2	2.18E-03	0.6
RHBDL2	rhomboid, veinlet-like 2 (Drosophila)	1.10E-03	0.6
ERMN	ermin, ERM-like protein, transcript variant 2	1.26E-02	0.6
DMBT1	deleted in malignant brain tumors 1, transcript variant 2	4.18E-02	0.6

Supplemental Table 3C. Top 50 upregulated genes in chronic active PL-NAWM vs inactive PL-NAWM (comparison II)

Gene symbol	Gene name	Adjusted *p*-value	Fold change
NPY	neuropeptide Y	1.15E-03	5.9
GABRA1	gamma-aminobutyric acid (GABA) A receptor, alpha 1, transcript variant 3	5.86E-03	3.9
SYNPR	synaptoporin, transcript variant 2	3.09E-03	3.8
NPTX1	neuronal pentraxin I	4.78E-02	3.7
VSNL1	visinin-like 1	3.72E-02	3.7
OPALIN	oligodendrocytic myelin paranodal and inner loop protein, transcript variant 3	7.36E-04	3.6
SYN2	synapsin II, transcript variant IIb	3.75E-02	3.5
SNAP25	synaptosomal-associated protein, 25kDa, transcript variant 1	4.47E-02	3.4
CREG2	cellular repressor of E1A-stimulated genes 2	2.12E-03	3.3
GABRB2	gamma-aminobutyric acid (GABA) A receptor, beta 2, transcript variant 1	1.36E-02	3.2
SYT4	synaptotagmin IV	1.17E-02	3.1
SYT13	synaptotagmin XIII	2.77E-02	3.1
NCAN	neurocan	1.98E-05	2.7
FGF13	fibroblast growth factor 13, transcript variant 1	2.49E-02	2.6
GDA	guanine deaminase	3.04E-02	2.6
SV2B	synaptic vesicle glycoprotein 2B, transcript variant 1	2.53E-02	2.5
CADPS	Ca++-dependent secretion activator, transcript variant 3	2.98E-03	2.5
BASP1	brain abundant, membrane attached signal protein 1	9.23E-03	2.5
MAL2	mal, T-cell differentiation protein 2	2.84E-02	2.5
GNLY	granulysin, transcript variant NKG5	1.72E-03	2.5
KCNC2	potassium voltage-gated channel, Shaw-related subfamily, member 2, transcript variant 1	4.97E-02	2.5
KCNMA1	potassium large conductance calcium-activated channel, subfamily M, alpha member 1, transcript variant 1	4.47E-02	2.4
CDH18	cadherin 18, type 2	4.70E-02	2.4
TNFRSF12A	tumor necrosis factor receptor superfamily, member 12A	9.98E-03	2.3
RGS7BP	regulator of G-protein signaling 7 binding protein	3.34E-02	2.3
CBLN2	cerebellin 2 precursor	3.44E-02	2.3
ENC1	ectodermal-neural cortex (with BTB-like domain)	3.03E-02	2.2
PRICKLE1	prickle homolog 1 (Drosophila), transcript variant 1	1.99E-03	2.2
MRAP2	melanocortin 2 receptor accessory protein 2	1.17E-02	2.2
HOPX	HOP homeobox (HOPX), transcript variant 2	1.37E-02	2.2
ELMOD1	ELMO/CED-12 domain containing 1, transcript variant 1	4.36E-02	2.2
FGF12	fibroblast growth factor 12, transcript variant 2	1.92E-02	2.2
CHI3L1	chitinase 3-like 1 (cartilage glycoprotein-39)	3.31E-02	2.1
NCEH1	neutral cholesterol ester hydrolase 1, transcript variant 2	1.20E-02	2.1
GABRB3	gamma-aminobutyric acid (GABA) A receptor, beta 3, transcript variant 1	2.34E-02	2.1
CD83	CD83 molecule, transcript variant 1	9.92E-03	2.1
ISG15	ISG15 ubiquitin-like modifier	2.25E-03	2.1
LRRN3	leucine rich repeat neuronal 3, transcript variant 3	5.48E-03	2.1
ALOX5AP	arachidonate 5-lipoxygenase-activating protein	1.27E-02	2.1
ADORA3	adenosine A3 receptor, transcript variant 1	5.58E-03	2.1
OLR1	oxidized low density lipoprotein (lectin-like) receptor 1 (OLR1)	4.36E-02	2.1
ANO4	anoctamin 4	8.29E-04	2.1
GAP43	growth associated protein 43, transcript variant 2	4.75E-02	2.1
GFRA2	GDNF family receptor alpha 2, transcript variant 1	3.22E-02	2.1
TMEM233	transmembrane protein 233 (TMEM233)	8.27E-03	2.1
MX1	myxovirus (influenza virus) resistance 1, interferon-inducible protein p78 (mouse), transcript variant 2	1.90E-03	2.0
CNR1	cannabinoid receptor 1 (brain), transcript variant 2	1.40E-02	2.0
BEX1	brain expressed, X-linked 1	4.88E-03	2.0
GPR98	G protein-coupled receptor 98, transcript variant 1	1.95E-02	2.0
IFI30	interferon, gamma-inducible protein 30	2.04E-02	2.0

Supplemental Table 3D. Top 50 downregulated genes in chronic active PL-NAWM vs inactive PL-NAWM (comparison II)

**Gene symbol**	**Gene name**	**Adjusted *p*-value**	**Fold change**
LOC100289290	PREDICTED: Homo sapiens similar to hCG2042717	4.21E-02	0.3
CRLF1	cytokine receptor-like factor 1	2.88E-02	0.3
IGJ	immunoglobulin J polypeptide, linker protein for immunoglobulin alpha and mu polypeptides	2.53E-02	0.3
ABCA6	ATP-binding cassette, sub-family A (ABC1), member 6	2.91E-02	0.3
KANK4	KN motif and ankyrin repeat domains 4	5.65E-06	0.3
ENST00000392423	titin isoform novex-3	1.13E-04	0.4
C21orf130	chromosome 21 open reading frame 130, non-coding RNA	5.99E-03	0.4
ARRDC4	arrestin domain containing 4	6.33E-03	0.4
ENST00000313339	Ankyrin repeat domain-containing protein 18A	3.61E-04	0.4
TSPAN8	tetraspanin 8	1.24E-04	0.4
TTN	titin, transcript variant N2-A	4.23E-03	0.4
ARMC3	armadillo repeat containing 3	4.78E-02	0.4
RBM11	RNA binding motif protein 11	6.76E-05	0.5
FSTL5	follistatin-like 5, transcript variant 1	2.54E-03	0.5
DHCR24	24-dehydrocholesterol reductase	1.34E-03	0.5
C15orf51	chromosome 15 open reading frame 51, non-coding RNA	8.10E-03	0.5
HYDIN	hydrocephalus inducing homolog (mouse), transcript variant 1	5.06E-04	0.5
ANKRD20B	ankyrin repeat domain 20B, non-coding RNA	9.18E-04	0.5
POU2AF1	POU class 2 associating factor 1	1.04E-02	0.5
MTUS1	microtubule associated tumor suppressor 1, transcript variant 2	6.76E-05	0.5
PPEF1	protein phosphatase, EF-hand calcium binding domain 1, transcript variant 1	1.62E-02	0.5
MBOAT1	membrane bound O-acyltransferase domain containing 1	3.09E-03	0.5
RELL1	RELT-like 1, transcript variant 1	6.64E-05	0.5
DPYSL5	dihydropyrimidinase-like 5	9.35E-04	0.5
LOC728449	Putative uncharacterized protein ENSP00000334090	7.27E-03	0.5
HN1L	hematological and neurological expressed 1-like	1.15E-03	0.5
TNFRSF21	tumor necrosis factor receptor superfamily, member 21	7.39E-03	0.5
ANKRD18A	PREDICTED: Homo sapiens ankyrin repeat domain 18A	1.00E-03	0.5
SNX24	sorting nexin 24	5.52E-04	0.5
SPARC	secreted protein, acidic, cysteine-rich (osteonectin)	4.14E-02	0.6
GOLGA8E	golgi autoantigen, golgin subfamily a, 8E	9.58E-03	0.6
LOC283481	hypothetical protein	7.39E-03	0.6
AK058117	cDNA FLJ25388 fis, clone TST02351	8.65E-03	0.6
ENST00000423618	Ankyrin repeat domain-containing protein 18B	1.10E-04	0.6
TTC25	tetratricopeptide repeat domain 25	4.52E-02	0.6
LOC645321	PREDICTED: Homo sapiens hypothetical LOC645321	1.05E-02	0.6
MRO	maestro, transcript variant 1	1.28E-03	0.6
KLK6	kallikrein-related peptidase 6, transcript variant B	8.19E-03	0.6
LOC284232	ankyrin repeat domain 20 family, member A2 pseudogene, non-coding RNA	1.44E-02	0.6
RND2	Rho family GTPase 2	8.65E-03	0.6
LRAT	lecithin retinol acyltransferase (phosphatidylcholine--retinol O-acyltransferase)	3.02E-03	0.6
NIPAL4	NIPA-like domain containing 4	9.58E-03	0.6
DNAJC15	DnaJ (Hsp40) homolog, subfamily C, member 15	1.07E-02	0.6
CCDC11	coiled-coil domain containing 11	5.52E-03	0.6
HAPLN2	hyaluronan and proteoglycan link protein 2	2.53E-02	0.6
OFD1	oral-facial-digital syndrome 1	2.52E-03	0.6
MAP4	microtubule-associated protein 4, transcript variant 4	1.95E-02	0.6
CFH	complement factor H, transcript variant 2	1.72E-03	0.6
DNAH12	dynein, axonemal, heavy chain 12, transcript variant 1	3.34E-02	0.6
MOBKL2B	MOB1, Mps One Binder kinase activator-like 2B (yeast)	3.86E-02	0.6

Supplemental Table 3E. Top 50 upregulated genes in chronic active PL-NAWM vs control WM (comparison III)

Gene symbol	Gene name	Adjusted *p*-value	Fold change
GPNMB	glycoprotein (transmembrane) nmb	7.40E-06	7.1
SERPINA3	serpin peptidase inhibitor, clade A (alpha-1 antiproteinase, antitrypsin), member 3	7.76E-04	7.0
APOC1	apolipoprotein C-I	3.21E-08	6.3
VIM	vimentin	1.07E-06	5.1
MSR1	macrophage scavenger receptor 1	1.64E-04	4.8
CD44	CD44 molecule (Indian blood group)	2.31E-03	4.5
OLR1	oxidized low density lipoprotein (lectin-like) receptor 1	4.86E-06	4.2
PALLD	palladin, cytoskeletal associated protein, transcript variant 2	5.95E-06	4.1
CHI3L1	chitinase 3-like 1 (cartilage glycoprotein-39)	2.63E-03	4.0
ANXA1	annexin A1	3.34E-02	3.9
EMP1	epithelial membrane protein 1	9.09E-03	3.8
S100A10	S100 calcium binding protein A10	1.72E-03	3.8
RFTN1	raftlin, lipid raft linker 1	6.08E-04	3.6
IFI30	interferon, gamma-inducible protein 30	2.08E-03	3.5
ANO6	anoctamin 6	2.46E-05	3.5
CP	ceruloplasmin (ferroxidase)	4.50E-04	3.5
ABCA1	ATP-binding cassette, sub-family A, member 1	5.18E-04	3.4
FPR3	formyl peptide receptor 3	1.34E-03	3.3
PLA2G7	phospholipase A2, group VII (platelet-activating factor acetylhydrolase, plasma)	6.16E-04	3.3
CD84	CD84 molecule	4.39E-05	3.3
TGFB2	transforming growth factor, beta 2, transcript variant 2	6.46E-05	3.1
FRMD3	FERM domain containing 3	4.76E-04	3.1
S100A6	S100 calcium binding protein A6	3.39E-05	3.1
NSL1	NSL1, MIND kinetochore complex component, homolog (S. cerevisiae), transcript variant 1	7.01E-11	3.0
LGALS3	lectin, galactoside-binding, soluble, 3, transcript variant 1	7.15E-05	3.0
SAMSN1	SAM domain, SH3 domain and nuclear localization signals 1	2.81E-03	3.0
MS4A7	membrane-spanning 4-domains, subfamily A, member 7, transcript variant 1	4.51E-02	3.0
COLEC12	collectin sub-family member 12	3.71E-04	2.9
DTNA	dystrobrevin, alpha, transcript variant 3	1.26E-04	2.9
FGF2	fibroblast growth factor 2 (basic)	3.16E-05	2.9
NCF2	neutrophil cytosolic factor 2, transcript variant 1	7.06E-03	2.9
SPP1	secreted phosphoprotein 1, transcript variant 1	1.58E-02	2.9
MAFB	v-maf musculoaponeurotic fibrosarcoma oncogene homolog B (avian)	1.78E-02	2.8
SORBS1	sorbin and SH3 domain containing 1, transcript variant 3	1.75E-05	2.8
DST	dystonin, transcript variant 1eB	8.96E-09	2.8
PTPRC	protein tyrosine phosphatase, receptor type, C, transcript variant 1	3.79E-04	2.8
DAAM1	dishevelled associated activator of morphogenesis 1	2.10E-04	2.8
DDR2	discoidin domain receptor tyrosine kinase 2, transcript variant 1	6.43E-05	2.8
TRIM34	tripartite motif-containing 34, transcript variant 3	1.10E-05	2.7
S1PR3	sphingosine-1-phosphate receptor 3	1.06E-03	2.7
DENND2D	DENN/MADD domain containing 2D	1.20E-02	2.7
TPST1	tyrosylprotein sulfotransferase 1	1.80E-04	2.6
MAN2A1	mannosidase, alpha, class 2A, member 1	2.32E-03	2.6
CNN3	calponin 3, acidic	2.26E-03	2.6
MAN1C1	mannosidase, alpha, class 1C, member 1	2.33E-03	2.6
CCRL2	chemokine (C-C motif) receptor-like 2, transcript variant 1	3.69E-04	2.6
EVI2B	ecotropic viral integration site 2B	1.55E-04	2.5
LIMS1	LIM and senescent cell antigen-like domains 1	5.63E-06	2.5
GPR65	G protein-coupled receptor 65	1.17E-02	2.5
SLC5A3	solute carrier family 5 (sodium/myo-inositol cotransporter), member 3	1.13E-03	2.5

Supplemental Table 3F. Top 50 downregulated genes in chronic active PL-NAWM vs control WM (comparison III)

Gene symbol	Gene name	Adjusted *p*-value	Fold change
AL050203	cDNA DKFZp586F1123 (from clone DKFZp586F1123)	1.05E-13	0.1
DEFA3	defensin, alpha 3, neutrophil-specific	9.85E-04	0.1
LOC100132168	hypothetical LOC100132168	7.01E-12	0.2
MYH7B	myosin, heavy chain 7B, cardiac muscle, beta	4.31E-13	0.2
ENST00000443002	DNA-directed RNA polymerases I, II, and III subunit	7.85E-16	0.2
CPAMD8	C3 and PZP-like, alpha-2-macroglobulin domain containing 8	1.46E-02	0.2
YJEFN3	YjeF N-terminal domain containing 3, nuclear gene encoding mitochondrial protein	2.02E-04	0.3
ATPGD1	ATP-grasp domain containing 1, transcript variant 1	3.26E-07	0.3
CIRBP	cold inducible RNA binding protein, transcript variant 2	3.03E-10	0.3
KCNQ2	potassium voltage-gated channel, KQT-like subfamily, member 2, transcript variant 5	5.84E-03	0.3
A_33_P3253832	Unknown	5.69E-06	0.3
ABCA2	ATP-binding cassette, sub-family A (ABC1), member 2, transcript variant 1	1.51E-11	0.3
CITED4	Cbp/p300-interacting transactivator, with Glu/Asp-rich carboxy-terminal domain, 4	1.67E-08	0.3
UNC84B	unc-84 homolog B (C. elegans)	5.56E-10	0.3
LOC283999	hypothetical protein LOC283999	1.49E-17	0.3
LIPE	lipase, hormone-sensitive (LIPE)	1.97E-07	0.3
ENST00000369123	Chromosome 6 open reading frame 220 Fragment	1.30E-06	0.3
CERCAM	cerebral endothelial cell adhesion molecule	2.30E-04	0.3
ENST00000293201	Putative myosin-XVB (Unconventional myosin-15B)(Myosin XVBP)	1.08E-08	0.3
LOC349114	hypothetical LOC349114 (LOC349114), non-coding RNA	1.75E-12	0.3
KLC2	kinesin light chain 2, transcript variant 2	5.17E-05	0.3
ENST00000405068	Exocyst complex component 7 (Exocyst complex component Exo70)	3.64E-08	0.3
DNAJB2	DnaJ (Hsp40) homolog, subfamily B, member 2, transcript variant 1	5.08E-13	0.3
ADORA1	adenosine A1 receptor, transcript variant 1	2.94E-12	0.3
LOC340335	cDNA FLJ23879 fis, clone LNG13743	4.79E-05	0.3
CCDC85B	coiled-coil domain containing 85B	9.80E-09	0.3
A_33_P3209176	Unknown	3.01E-05	0.3
ENST00000371623	Prostaglandin-H2 D-isomerase Precursor (EC 5.3.99.2)	2.18E-16	0.3
PCBP4	poly(rC) binding protein 4, transcript variant 4	1.53E-17	0.3
STMN4	stathmin-like 4	2.68E-02	0.3
CMTM5	CKLF-like MARVEL transmembrane domain containing 5, transcript variant 3	1.02E-08	0.3
TMEM63A	transmembrane protein 63A	4.41E-08	0.3
DAO	D-amino-acid oxidase (DAO)	8.86E-03	0.3
HMX1	H6 family homeobox 1	9.00E-09	0.3
A_33_P3277805	Unknown	1.52E-02	0.3
LTBP3	latent transforming growth factor beta binding protein 3, transcript variant 2	1.57E-10	0.3
DOHH	deoxyhypusine hydroxylase/monooxygenase, transcript variant 2	1.67E-07	0.3
FTCD	formiminotransferase cyclodeaminase, transcript variant A	4.36E-05	0.3
ST3GAL4	ST3 beta-galactoside alpha-2,3-sialyltransferase 4	5.47E-10	0.3
FLJ45445	hypothetical LOC399844, non-coding RNA	1.57E-10	0.3
PAQR6	progestin and adipoQ receptor family member VI, transcript variant 1	1.19E-10	0.3
tcag7.907	hypothetical LOC402483 (FLJ45340), non-coding RNA	2.99E-03	0.3
PNPLA7	patatin-like phospholipase domain containing 7, transcript variant 1	6.75E-07	0.3
LONP1	lon peptidase 1, mitochondrial, nuclear gene encoding mitochondrial protein	3.62E-15	0.3
SBF1	SET binding factor 1	1.30E-10	0.3
ENST00000401999	hypothetical LOC401357	1.52E-09	0.3
HES6	hairy and enhancer of split 6 (Drosophila), transcript variant 1	6.99E-13	0.3
AGAP3	ArfGAP with GTPase domain, ankyrin repeat and PH domain 3, transcript variant 2	9.13E-07	0.3
ATP6V0E2	ATPase, H+ transporting V0 subunit e2, transcript variant 1	1.14E-07	0.3
MVD	mevalonate (diphospho) decarboxylase	2.19E-15	0.3
