# Supplementary material for: Gene Expression Profiling of Multiple Sclerosis Pathology Identifies Early Patterns of Demyelination Surrounding Chronic Active Lesions
Source: Front Immunol. 2017 Dec 21;8:1810. doi: 10.3389/fimmu.2017.01810 (PMC5742619; doi:10.3389/fimmu.2017.01810)
Supplement: Supplementary file 6 [file Table_4.PDF]

Supplemental Table 4. Gene ontology clusters

Term	GO class	Count	Total	*p*-value
**Cluster 1**				
biological process				
ion transport	GO:0006811	46	732	3.46E-03
cell junction	GO:0030054	28	370	3.46E-03
small GTPase mediated signal transduction	GO:0007264	29	397	3.83E-03
sphingomyelin catabolic process	GO:0006685	3	3	6.40E-03
transport	GO:0006810	109	2317	7.90E-03
localization	GO:0051179	123	2718	1.11E-02
monovalent inorganic cation transport	GO:0015672	22	298	1.19E-02
establishment of localization	GO:0051234	110	2401	1.37E-02
cellular component				
microtubule	GO:0005874	18	203	4.48E-03
anchoring collagen	GO:0030934	4	9	1.19E-02
synaptic vesicle	GO:0008021	8	49	1.38E-02
molecular function				
purine ribonucleotide binding	GO:0032555	82	1580	3.83E-03
ribonucleotide binding	GO:0032553	82	1580	3.83E-03
MAP-kinase scaffold activity	GO:0005078	3	3	6.40E-03
purine nucleotide binding	GO:0017076	83	1650	6.95E-03
passive transmembrane transporter activity	GO:0022803	26	371	1.10E-02
channel activity	GO:0015267	26	371	1.10E-02
GTPase activator activity	GO:0005096	15	168	1.10E-02
ion channel activity	GO:0005216	25	354	1.13E-02
substrate specific channel activity	GO:0022838	25	362	1.38E-02
GTPase activity	GO:0003924	15	175	1.42E-02
**Cluster 2**				
biological process				
immune system process	GO:0002376	50	715	3.39E-09
immune response	GO:0006955	39	544	2.25E-07
cell proliferation	GO:0008283	42	691	4.14E-05
response to external stimulus	GO:0009605	35	571	3.67E-04
developmental process	GO:0032502	118	2889	3.69E-04
hematopoiesis	GO:0030097	17	154	3.69E-04
immune system development	GO:0002520	18	173	3.69E-04
hematopoietic or lymphoid organ development	GO:0048534	17	165	7.32E-04
regulation of cell proliferation	GO:0042127	28	437	7.87E-04
anatomical structure development	GO:0048856	81	1861	1.28E-03
organ development	GO:0048513	53	1090	2.07E-03
response to wounding	GO:0009611	24	381	5.16E-03
regulation of multicellular organismal process	GO:0051239	18	252	5.49E-03
defense response	GO:0006952	29	505	6.36E-03
positive regulation of cell proliferation	GO:0008284	16	216	6.94E-03
system development	GO:0048731	66	1521	7.91E-03
immune effector process	GO:0002252	11	97	7.91E-03
B cell activation	GO:0042113	8	54	1.03E-02
cell death	GO:0008219	38	757	1.03E-02
death	GO:0016265	38	757	1.03E-02
regulation of immune system process	GO:0002682	10	86	1.03E-02
cell activation	GO:0001775	14	186	1.07E-02
inflammatory process	GO:0006954	18	271	1.07E-02
cytoskeletal protein binding	GO:0008092	23	385	1.07E-02
regulation of programmed cell death	GO:0043067	27	482	1.07E-02
programmed cell death	GO:0012501	36	716	1.21E-02
negative regulation of programmed cell death	GO:0043069	15	210	1.24E-02
nucleosome positioning	GO:0016584	3	5	1.29E-02
myeloid cell differentiation	GO:0030099	9	75	1.29E-02
response to stress	GO:0006950	44	940	1.36E-02
chemotaxis	GO:0006935	12	130	1.37E-02
taxis	GO:0042330	12	130	1.37E-02
multicellular organismal development	GO:0007275	82	2068	1.49E-02
apoptosis	GO:0006915	35	710	1.85E-02
regulation of apoptosis	GO:0042981	26	477	1.85E-02
B cell receptor signaling pathway	GO:0050853	3	6	1.98E-02

cell morphogenesis	GO:0000902	23	408	2.13E-02
cellular structure morphogenesis	GO:0032989	23	408	2.13E-02
regulation of immune response	GO:0050776	9	84	2.13E-02
response to virus	GO:0009615	9	84	2.13E-02
adaptive immune response	GO:0002250	8	69	2.48E-02
adaptive immune response based on somatic recombination of immune receptors built from immunoglobulin superfamily domains	GO:0002460	8	69	2.48E-02
production of molecular mediator of immune response	GO:0002440	5	26	2.58E-02
regulation of binding	GO:0051098	5	26	2.58E-02
regulation of biological process	GO:0050789	141	4043	2.63E-02
cellular developmental process	GO:0048869	65	1598	2.63E-02
cell differentiation	GO:0030154	65	1598	2.63E-02
cell growth	GO:0016049	13	167	2.63E-02
leukocyte mediated immunity	GO:0002443	8	71	2.63E-02
positive regulation of immune response	GO:0050778	8	72	2.76E-02
negative regulation of mast cell cytokine production	GO:0032764	2	2	2.76E-02
regulation of germinal center formation	GO:0002634	2	2	2.76E-02
negative regulation of apoptosis	GO:0043066	14	208	2.81E-02
negative regulation of biological process	GO:0048519	46	1046	2.86E-02
positive regulation of immune system response	GO:0002684	8	73	2.86E-02
cell development	GO:0048468	47	1076	2.87E-02
regulation of cell size	GO:0008361	13	172	2.98E-02
positive regulation of biological process	GO:0048518	43	967	3.01E-02
immunoglobulin production	GO:0002377	4	17	3.01E-02
chitin catabolic process	GO:0006032	3	8	3.01E-02
N-acetylglucosamine catabolic process	GO:0006046	3	8	3.01E-02
regulation of mRNA stability	GO:0043488	3	8	3.01E-02
chitin metabolic process	GO:0006030	3	8	3.01E-02
chitinase activity	GO:0004568	3	8	3.01E-02
regulation of RNA stability	GO:0043487	3	8	3.01E-02
positive regulation of B cell proliferation	GO:0030890	3	8	3.01E-02
biological regulation	GO:0065007	152	4458	3.01E-02
regulation of cell motility	GO:0051270	7	59	3.07E-02
response to chemical stimulus	GO:0042221	26	506	3.19E-02
anatomical structure morphogenesis	GO:0009653	42	947	3.34E-02
regulation of biological quality	GO:0065008	35	753	3.55E-02
regulation of developmental process	GO:0050793	14	217	3.83E-02
regulation of locomotion	GO:0040012	7	62	3.83E-02
amino sugar catabolic process	GO:0046348	3	9	3.86E-02
glycosamine catabolic process	GO:0006043	3	9	3.86E-02
regulation of B cell proliferation	GO:0030888	3	9	3.86E-02
locomotion	GO:0040011	7	63	3.96E-02
leukocyte activation	GO:0045321	12	164	4.47E-02
erythrocyte differentiation	GO:0030218	5	33	4.57E-02
locomotory behavior	GO:0007626	12	165	4.60E-02
negative regulation of immune system process	GO:0002683	3	10	4.81E-02
regulation of Rho GTPase activity	GO:0032319	3	10	4.81E-02
negative regulation of cytokine production during immune response	GO:0002719	2	3	4.81E-02
negative regulation of production of molecular mediator of immune response	GO:0002701	2	3	4.81E-02
cellular component				
basement membrane	GO:0005604	9	63	6.94E-03
extracellular matrix part	GO:0044420	10	95	1.49E-02
plasma membrane part	GO:0044459	69	1681	1.71E-02
plasma membrane	GO:0005886	106	2859	2.13E-02
proteinaceous extracellular matrix	GO:0005578	18	304	3.01E-02
cytoplasm	GO:0005737	180	5493	4.81E-02
molecular function				
protein binding	GO:0005515	214	6395	9.65E-03
actin binding	GO:0003779	18	265	9.77E-03
GTPase binding	GO:0051020	9	71	1.07E-02
small GTPase binding	GO:0031267	7	63	3.96E-02
transcription corepressor activity	GO:0003714	9	103	4.78E-02
mannosyl-oligosaccharide mannosidase activity	GO:0015924	3	10	4.81E-02
signal transducer activity	GO:0004871	77	2039	4.81E-02
molecular transducer activity	GO:0060089	77	2039	4.81E-02
lipopolysaccharide binding	GO:0001530	2	3	4.81E-02
**Cluster 4**				

biological process				
antigen processing and presentation of peptide or polysaccharide antigen via MHC class II	GO:0002504	10	16	2.06E-16
immune response	GO:0006955	27	544	1.25E-15
antigen processing and presentation	GO:0019882	12	53	3.06E-14
immune system response	GO:0002376	28	715	4.07E-14
response to biotic stimulus	GO:0009607	13	242	1.36E-07
defense response	GO:0006952	17	505	3.24E-07
response to other organism	GO:0051707	11	177	4.36E-07
multi-organism process	GO:0051704	13	279	5.08E-07
response to virus	GO:0009615	8	84	1.83E-06
inflammatory response	GO:0006954	9	271	1.08E-03
humoral immune response	GO:0006959	5	65	1.42E-03
antigen processing and presentation of peptide antigen via MHC class I	GO:0002474	3	12	1.49E-03
response to wounding	GO:0009611	10	381	2.51E-03
antigen processing and presentation of peptide antigen	GO:0048002	3	15	2.85E-03
response to external stimulus	GO:0009605	11	571	1.34E-02
response to chemical stimulus	GO:0042221	10	506	1.87E-02
response to stress	GO:0006950	13	940	4.02E-02
cellular component				
MHC protein complex	GO:0042611	11	33	6.17E-15
MHC class II protein complex	GO:0042613	9	15	6.17E-15
plasma membrane part	GO:0044459	33	1681	1.35E-12
lysosome	GO:0005764	12	179	5.49E-08
lytic vacuole	GO:0000323	12	179	5.49E-08
vacuole	GO:0005773	12	199	1.51E-07
plasma membrane	GO:0005886	39	2859	1.87E-07
integral to plasma membrane	GO:0005887	20	1152	1.28E-05
intrinsic to plasma membrane	GO:0031226	20	1168	1.75E-05
lysosomal membrane	GO:0005765	5	45	3.10E-04
membrane	GO:0016020	56	6035	4.39E-04
vacuolar membrane	GO:0005774	5	54	7.01E-04
vacuolar part	GO:0044437	5	56	7.46E-04
membrane part	GO:0044425	47	5112	4.80E-03
integral to membrane	GO:0016021	42	4488	9.50E-03
intrinsic to membrane	GO:0031224	42	4509	1.05E-02
pigment granule	GO:0048770	4	83	3.49E-02
melanosome	GO:0042470	4	83	3.49E-02
molecular function				
MHC class II receptor activity	GO:0032395	4	7	2.24E-06
signal transducer activity	GO:0004871	26	2039	7.46E-04
molecular transducer activity	GO:0060089	26	2039	7.46E-04
receptor activity	GO:0004872	21	1607	3.38E-03
IgG binding	GO:0019864	2	6	1.43E-02
tumor necrosis factor binding	GO:0043120	2	9	3.14E-02
C-X-C chemokine binding	GO:0019958	2	10	3.54E-02
MHC class I receptor activity	GO:0032393	2	10	3.54E-02
**Cluster 5**				
biological process				
skeletal development	GO:0001501	3	204	2.81E-02
phosphate transport	GO:0006817	2	87	4.31E-02
cellular component				
collagen type I	GO:0005584	2	3	5.45E-04
fibrillar collagen	GO:0005583	2	11	3.31E-03
extracellular matrix part	GO:0044420	3	95	5.23E-03
collagen	GO:0005581	2	34	1.96E-02
extracellular region part	GO:0044421	5	741	1.96E-02
proteinaceous extracellular matrix	GO:0005578	3	304	4.31E-02
muscle thin filament tropomyosin	GO:0005862	1	4	4.53E-02
microfibril	GO:0001527	1	4	4.53E-02
molecular function				
structural constituent of bone	GO:0008147	2	4	5.45E-04
thyroid hormone transmembrane transporter activity	GO:0015349	1	2	3.51E-02
prothoracicotrophic hormone activity	GO:0018445	1	2	3.51E-02
interleukin-7 binding	GO:0019982	1	2	3.51E-02
interleukin-7 receptor activity	GO:0004917	1	2	3.51E-02
cytokine binding	GO:0019955	2	85	4.31E-02

extracelluar matrix structural constituent	GO:0005201	2	87	4.31E-02
**Cluster 6**				
biological process				
sterol biosynthetic process	GO:0016126	2	32	4.30E-02
plasminogen activation	GO:0031639	1	1	4.65E-02
steroid biosynthetic process	GO:0006694	2	74	4.65E-02
sterol metabolic process	GO:0016125	2	80	4.65E-02
molecular function				
squalene monooxygenase activity	GO:0004506	1	1	4.65E-02
Rap guanyl-nucleotide exchange factor activity	GO:0017034	1	2	4.65E-02

Clusters are specified in Figure 2. Count = number of significantly regulated genes within GO class; Total = total number of genes within GO class.
